# Health outcomes, health services utilization, and costs consequences of medicare uninsurance among migrants in Canada: a systematic review

**DOI:** 10.1186/s12913-023-09417-4

**Published:** 2023-05-03

**Authors:** Sophiya Garasia, Valerie Bishop, Stephanie Clayton, Genevieve Pinnington, Chika Arinze, Ezza Jalil

**Affiliations:** 1 Department of Health Research Methods, Evidence, and Impact, McMaster University, 1280 Main Street West, Hamilton, ON L8S 4K1 Canada; 2 Centre for Health Economics and Policy Analysis, McMaster University, Hamilton, ON Canada; 3 Department of Oncology, McMaster University, Hamilton, ON Canada

**Keywords:** Migrant, Health, Medically uninsured, Health services, Canada

## Abstract

**Background:**

Medically uninsured groups, many of them migrants, reportedly delay using healthcare services due to costs and often face preventable health consequences. This systematic review sought to assess quantitative evidence on health outcomes, health services use, and health care costs among uninsured migrant populations in Canada.

**Methods:**

OVID MEDLINE, Embase, Global Health, EconLit, and grey literature were searched to identify relevant literature published up until March 2021. The Cochrane Risk of Bias in Non-randomized Studies – of Interventions (ROBINS-I) tool was used to assess the quality of studies.

**Results:**

Ten studies were included. Data showed that there are differences among insured and uninsured groups in reported health outcomes and health services use. No quantitative studies on economic costs were captured.

**Conclusions:**

Our findings indicate a need to review policies regarding accessible and affordable health care for migrants. Increasing funding to community health centers may improve service utilization and health outcomes among this population.

**Supplementary Information:**

The online version contains supplementary material available at 10.1186/s12913-023-09417-4.

## Background

### Canadian health care

Despite having “universal” medical care coverage for physicians and hospitals in Canada, there are many residents who remain ineligible. The number of uninsured individuals is estimated to be between 200,000 to 500,000 people in Ontario alone [1]. The exact number is unknown given the difficulties in collecting data from this population. The impact of lack of health care insurance on these individuals, who are mainly migrants, is an understudied area in Canada to-date [2–6].

On average, over 300,000 new international migrants come to Canada annually and this number continues to increase [7]. The United Nations Migration Agency defines a migrant as someone who is moving or has moved across an international border or within a state away from their place of birth [8]. From January to March 2019, 82.0% of Canada’s population growth came from international migration [7].

Canada has a publicly funded health care system, Canadian Medicare, which provides residents free access to medically necessary hospital care and physician services [9, 10]. Instead of having a single national plan, Canadian Medicare is comprised of thirteen provincial and territorial health care insurance plans [10]. Residents receive medical coverage from the province or territory that they live in. The Federal government contributes to the financing of provincial/territorial medical health care systems provided that the provinces/territories adhere to the principles of the Canada Health Act (i.e., public administration, comprehensiveness, universality, portability, accessibility). However, there are exceptions—the Interim Federal Health Program (IFHP) offers temporary coverage of basic health services for refugees and asylum seekers, and the Indian Act of 1876 gives the federal government responsibility for the health care of Indigenous Peoples living on reserves. Nevertheless, some individuals are not eligible for provincial medical insurance coverage due to a lack of permanent residence status which according to the Canada Health Act principle of “universality” is a must. As the definition of “resident” is left to the provinces and territories, the eligibility criteria varies across Canada [11]. In Quebec, Ontario, British Columbia, and Manitoba, a three-month waiting period is imposed on new permanent residents before they qualify for provincial medical insurance [12]. This policy was removed in Ontario during the Coronavirus pandemic [13] and it is unclear whether it will be reinstated. Further, undocumented and out-of-status migrants do not qualify for Canadian Medicare and in Ontario, it is estimated that there are approximately 250,000 out-of-status migrants who do not have access to health care coverage [14]. In other words, the uninsured population in Canada is quite heterogenous. At the same time, the migrant population is also heterogenous made up of economic class immigrants, international students, seasonal workers, and refugees, among many others [15]. In Canada, there is a strong relationship between migrant status and being medically uninsured [16].

Lack of insurance coverage can also interact with other determinants of health to influence the health and health care experiences of migrants. For example, without provincial medical coverage, migrants often rely on private insurance or make out-of-pocket payments which can be costly to low-income individuals [17]. Other factors including insufficient knowledge of the Canadian health care system, language barriers, fear of deportation, cultural differences, and discrimination or denial of care also generate barriers and contribute to a decline in health status [3, 5, 18, 19].

### Health and health care services use among uninsured

Reduced coverage for migrants may result in an increase in patients seeking emergency care as a last resort for conditions such as uncontrolled diabetes or mental health issues that could have been addressed at earlier stages [20]. Medically uninsured migrants with children often experience delays in surgeries, lack of adequate care for mental health issues, and inability to access support for developmental disabilities [4]. Uninsured pregnant women are also a group of concern as many go without any prenatal care and may experience complications during labour and delivery [4, 21]. These barriers to accessing care can lead to increased complications from untreated or delayed diagnoses of acute or chronic conditions [22]. Lack of insurance coverage among migrants has been associated with lower self-perceived health [23].

Extensive literature from high-income countries including Canada have focused on the “healthy immigrant effect” [24]. It states that immigrants on arrival are healthier than non-immigrants in the receiving country, but with time, their health deteriorates and resembles that of non-migrant residents. The reason why immigrants are healthy on arrival is said to be because of selection at both the individual (individuals who are healthy make the decision to migrate) and institutional (individuals who are healthy and who have high education, professional experience, and show potential to contribute to the Canadian economy are selected by the State) level. The reason why health deteriorates is also said to be because of a number of reasons (e.g., racism, lack of employment), one of which is the lack of health services usage [25, 26]. The strength of the healthy immigrant effect has shown to differ across groups, however [26, 27]. For instance, Lu and Ng (2019), using a Canadian linked dataset, found that the healthy immigrant effect on various health outcomes differed across immigrant categories [27]. It was stronger in economic-class immigrants while among refugees, it was only seen for less severe chronic conditions. Moreover, some studies challenge the healthy immigrant effect and instead provide results for the “sick immigrant effect” which states that immigrants are unhealthier than their native counterparts, even on arrival [28, 29]. This is sometimes the case with refugees who have less-strict guidelines for immigration since the main goal of refugee policies is to help those in dire circumstances.

### Financial impact of medical uninsurance

Reductions to IFHP in 2012, which have since been reinstated, were projected to save $50 million per year [22, 30]. However, a study conducted by Evans et al. (2014) found that these costs were ultimately transferred to hospitals, many of which have policies and ethical responsibilities to provide care in emergencies regardless of payment [30]. For example, the University Health Network in Toronto attributed over $800,000 in unpaid service debt to uninsured emergency services as a result of IFHP changes [20]. The three-month waiting period in Ontario was also implemented for cost-cutting purposes and to prevent individuals from entering the country solely for the purpose of utilizing “free” health care [31]. Although its economic and health impact is not clearly known, critics have mentioned that the policy may not be cutting costs as expected [1, 32, 33]. Individuals may be delaying necessary care until the wait period is over. Delaying care can increase health care system costs as people may be using health care services for conditions that have since worsened and are more severe and costly to treat in month four [17]. Hospital costs may also be transferred to community organizations who often provide care to those who do not have medical insurance while lacking the funds that hospitals have. At the individual level, individuals without medical insurance have to pay for primary or hospital care in Canada via private insurance or out-of-pocket. Not only is this a health equity issue, it can lead to these individuals facing financial problems which in turn could affect their health conditions even more. An understanding of what the literature shows in terms of out-of-pocket expenditures for the provincially medically uninsured and the costs to the health care system (public or private) to provide care to the medically uninsured would help evaluate policies targeted towards medically uninsured individuals.

Altogether there is limited understanding of the economic and health impact of Medicare uninsurance among migrants in Canada. Although challenges faced by migrants are widely known in Canada [14], at present there are limited reviews focusing on uninsured migrants and to our knowledge, no systematic reviews examining all migrant populations in Canada such as refugees, undocumented migrants, new permanent residents affected by the three-month waiting period, and international students [3, 5, 33]. Previous Canadian reviews have also focused on qualitative primary studies [5, 33]. As migration and the number of uninsured individuals increase, it is important to gain a comprehensive understanding of health outcomes and health care utilization trends among medically uninsured populations in Canada as well as understand the size of the problem. A systematic analysis of literature can inform where there are gaps in research as well as what the priority needs are. As such, this review sought to synthesize quantitative literature on health outcomes, health care utilization, and out-of-pocket public expenditures and/or costs to the public or private health care system to provide care to medically uninsured populations. Following systematic review methodology, this review also aimed to conduct quality assessment, which is also known as quality appraisal, critical appraisal, and risk of bias assessment. Although systematic reviews are often considered to be the highest level of evidence in the literature, they have their own biases that can lead to some studies having a greater weight in influencing the recommendations made in the review [34–36]. To prevent this, the methodological quality and rigor of each of the studies was assessed and reported on.

## Methods

### Research question and protocol

This systematic review asks: “What health outcomes, health care utilization trends, and health care costs are reported among uninsured migrant populations in Canada?” For the purpose of this review, with the understanding that the uninsured group is quite heterogenous in Canada, uninsured populations were broadly defined as individuals who are provincially medically uninsured (i.e., do not receive health care coverage from their province/territory for medically necessary physician and hospital services). This includes but is not limited to permanent residents waiting for their health card during the three month arrival period in certain provinces, undocumented individuals, asylum seekers who are denied or awaiting their refugee claim, refugees who are refused benefits under the IFHP, temporary foreign workers, visitors, and international students. A systematic review protocol was created a priori to ensure transparency, reproducibility, and consistency.

### Search strategy

A comprehensive search algorithm was created and implemented in Ovid MEDLINE, EconLit, Embase, and Global Health on March 9, 2021. The algorithm was created in consultation with a university librarian, after searching “uninsured AND Canada AND health” in MEDLINE and reviewing key words in the title and abstract of relevant papers until saturation was reached. The final search algorithm consisted of key terms related to “uninsured migrant”, “health”, and “Canada” and is further detailed in the supplementary document. To ensure the electronic database search captured all relevant literature, reference lists from all included studies and relevant reviews were also screened. In addition, targeted grey literature was searched on government and research organization websites. The first ten pages of Google Scholar were searched on March 30, 2021 using the following search terms: “uninsured AND Canada AND health”, following guidelines by Haddaway et al. (2015) [37].

### Inclusion and exclusion criteria

Articles were included if they a) studied the population of interest (uninsured migrants in Canada), b) examined the intervention of interest (Medicare uninsurance), c) reported at least one outcome of interest (health outcomes, health service utilization, or health care costs), and d) reported primary quantitative data. All reviews were excluded. Other exclusion criteria included notes, editorials, books, news reports, case reports, commentaries, opinions, and letters. Qualitative research was also excluded given that previous reviews have already summarized qualitative literature on this population in Canada [5, 16, 33]. In addition, we were interested in synthesizing the quantitative relationship between medical uninsurance and various outcomes (i.e., health outcomes, health care use, and cost) and understanding the size of the problems affecting the medically uninsured, for which quantitative research was relevant.

Studies were also excluded if they focused on a migrant population residing outside of Canada. Additionally, studies examining the lack of health insurance for services not covered under Canadian Medicare, such as dental or vision care, were excluded. Due to financial and human resource limitations, studies reported in languages other than English were excluded. Lastly, studies examining the IFHP were excluded because this federal program provides comparable health insurance coverage for some medical care services to refugees and refugee claimants (IFHP is intended to be comparable to the provincial medical care insurance programs. It provides coverage for basic health care services as well as supplemental services and prescription drug coverage), and thus does not meet the inclusion criteria [38].

### Screenings, extraction and quality assessment

Title and abstracts of papers were screened by two investigators independently using the inclusion and exclusion criteria. Disagreements were discussed and resolved between them. Full-text reviews were conducted for studies that could not be excluded on the basis of title and abstract content. Data extraction and quality assessment were conducted independently by two reviewers, and conflicts were managed by a third reviewer. Some information extracted from the studies included: year of publication, study location, demographic characteristics of the population, study design, uninsured definition, objectives of the study, outcome measures, and main results.

The Cochrane Risk of Bias in Non-randomized Studies – of Interventions (ROBINS-I) tool was used to assess the quality of included studies. The overall ratings for risk of bias were classified as low, moderate, serious, or critical. The tool was selected as it screens studies for different types of biases including selection, performance, detection, attrition, and reporting bias [39]. Utilizing ROBINS-I ensured that conclusions were formed while considering the quality of the studies [39].

### Review management

Citations were imported into Covidence, an online systematic review software. The software was used for de-duplication and relevance screening to screen the titles, abstracts, and full-texts of identified articles. Five reviewers took part in the screening process. All studies were screened between March 9, 2021, and March 30, 2021. Data was extracted from relevant papers and recorded in Microsoft Excel which was also used for descriptive analysis and charting. We utilized the Preferred Reporting Items for Systematic reviews and Meta-Analyses (PRISMA) statement to guide our reporting process [40].

## Results

### Search results

**Fig. 1:**
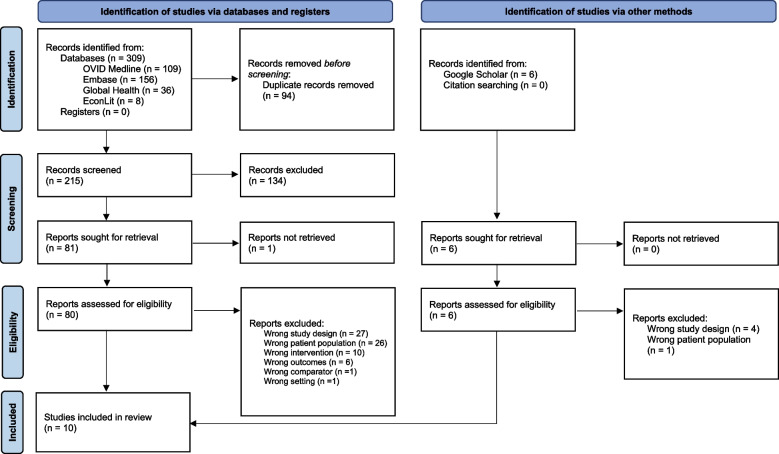
PRISMA representation of the search results

The search yielded 215 articles after deduplication. Of these, 134 were excluded at the title and abstract stage because they were not relevant to the topic of interest. At the full-text review stage, studies failing to meet the inclusion criteria for study design, patient population, intervention, outcomes, comparator group, and setting were excluded. An additional six studies were identified through a grey literature search of Google Scholar, of which one was included. A review of the reference lists of relevant scoping reviews yielded no additional studies. Two studies used the same data but their research questions and results slightly differed, and so were extracted and reported separately [22, 23]. A total of ten articles were included in the review. Figure 1 provides a complete overview of the study selection.

### Characteristics of included studies

Table 1 shows the characteristics of included studies. Studies were conducted in the metropolitan cities of Toronto [41–45], Montreal [22, 23, 42, 45, 46], and Vancouver [42] in the provinces of Ontario [45, 47, 48], Quebec [22, 23, 42, 45, 46], and British Columbia [42], respectively. Studies used data from 2002 to 2017, and were published between 2011 and 2020, with the majority being published in 2013 [41–43, 45]. Most of the studies used chart reviews or hospital administrative data and conducted retrospective analysis [41, 43–47]. Three cross-sectional studies based on questionnaires were also included [22, 23, 48], as well as one prospective cohort study [42] using a combination of a questionnaire and administrative data. Due to the nature of study designs, longitudinal analysis was not applicable for most studies. The majority of studies used data from hospitals, while three used data from the community [22, 23, 44].

**Table 1:** Characteristics of included studies

Author & year of publication	Study design (Accrual)	City and Province	Research question / study objectives	Data collection	Total sample size (n)	Limitations reported by authors
Cloos, Ndao, Aho, et al., (2020) [23]	Cross-sectional (2016 to 2017)	Montréal, Québec	To examine the association between precarious migration status and self-perceived health in Montréal	-Snowball sampling, local media campaign in community, and recruitment through health clinic -Face-to-face questionnaire -Subsequent focus groups	806	-Potentially unrepresentative sample -Recruiting uninsured in a clinic could introduce selection bias -Self-reporting could introduce social desirability bias -Potential for misreporting -Lack of power due to sample size -No control for confounding effects -Cross-sectional study design makes it difficult to establish causality
Ridde, Aho, Ndao, et al., (2020) [22]	Cross-sectional (2016 to 2017)	Montréal, Québec	To examine the unmet health care needs and its associated factors among Medicare uninsured migrants residing in Montréal	-Snowball sampling, local media campaign in community, and recruitment through health clinic -Face-to-face questionnaire -Subsequent focus groups	806	-Certain social groups (Chinese and Anglo-Caribbean migrants) were underrepresented -Participants could have been surveyed twice given that no personal information was collected to identify participants -Risk for interviewer bias is possible -Did not collect objective data (such as health outcomes) -Cross-sectional study design makes it difficult to establish causality
Darling, Bennett, Burton, et al., (2019) [47]	Retrospective cohort (2012 to 2015)	Ontario	To analyze the characteristics, health service utilization, and clinical outcomes of Ontario residents not covered by Ontario Health Insurance Plan (OHIP) that receive services from midwives	- BORN-Ontario records meeting criteria during specified time period (pregnancy to 6 weeks postpartum) for all midwifery clients	55,634	-Did not do chi-square analysis to see whether the proportions differ by insurance status -BORN-Ontario registry does not provide a reason why individuals are uninsured and whether they had non-OHIP insurance
Hynie, Ardern, & Robertson (2016) [48]	Cross-sectional (9 consecutive years, 2002/3 to 2010/11)	Ontario	To compare the diagnoses, severity, and outcomes associated with acute care visits by Ontario residents with and without insurance	-Data of all emergency visits in the National Ambulatory Care Reporting System (NACRS)	44,489,750 (unique emergency department visits)	-Repeat visits may have caused an increase in the number of uninsured -Those excluded due to homelessness could have been uninsured -NACRS data represent number of unique visits, not individuals -Cross-sectional study design makes it difficult to establish causality
Bunn, Fleming, Rzeznikiewiz, et al., (2013) [41]	Retrospective cohort (2005 to 2009)	Toronto, Ontario	To determine demographic and diagnostic information about the medically uninsured patient population and compare it with that of the medically insured patient population at a primary care centre	-Medical charts and billing records to determine number of uninsured individuals -Random sampling used to obtain insured individuals	95	-Lack of power due to sample size -Low external validity -Members of uninsured group were uninsured for a number of reasons; heterogeneous group -Unknown if all participants were screened for all of the diagnoses investigated -No control for confounding effects -Internal validity of this study was limited by the fact that medical charts of 7 uninsured patients and 2 insured patients could not be located -Unclear whether the two groups were comparable in all fronts except for insurance status; only compared income, age, and sex
Gagnon, Merry, & Haase (2013) [42]	Prospective cohort (2006 to 2009)	Toronto, Ontario, Montréal, Québec and Vancouver, British Columbia	To determine predictors (social, biomedical, migration, and health service) of emergency cesarean delivery in order to develop a better understanding of disparities in emergency cesarean delivery rates between Canadian-born and migrant women	-Convenience & alternate sampling -Recruited through the Childbearing Health and Related Services Needs of Newcomers study -Medical chart review and interviewer-assisted validated questionnaire	1,025	-Heterogeneity of comparison group -Uninsured population was not defined. Unclear whether sample included refugees, asylum-seekers, or immigrants -No analysis of maternity unit characteristics -Full regression results were not presented -Canadian-born women were included in the original study but they did not act as a comparator here
Rousseau, Laurin-Lamothe, Rummens, et al., (2013) [45]	Retrospective cohort (2008 to 2009)	Montréal, Québec and Toronto, Ontario	To examine the differences in help-seeking and service delivery across migratory statuses, institutions and provinces	-Chart review of patient records from 3 hospitals (2 in Montréal, 1 in Toronto) -Charts were randomly sampled from a curated list of uninsured files -Hospital 1 (Montréal) randomly selected 500 files for review -Hospital 2 (Montréal) reviewed all files (805) without a health insurance number -Hospital 3 (Toronto) reviewed 902 files (576 refugee claimants with IFHP coverage and 406 uninsured immigrant, refugee or undocumented patients without provincial coverage)	2,035	-Due to the retrospective chart review design, sociodemographic variables were unavailable or missing and could not be accounted for -No control for confounding effects -Potential differences across hospitals were not studied
Wilson-Mitchell & Rummens, (2013) [43]	Retrospective cohort (2007 to 2010)	Toronto, Ontario	To examine the relationship between insurance status and perinatal outcomes	-Chart review of hospital records -Insured patients were randomly selected -Uninsured patients were obtained from hospital record lists using self-pay payment codes	453	-Retrospective chart reviews may be inaccurate or inconsistent -Low external validity -Lack of power due to sample size -Researchers could not match uninsured to insured because demographic information was either inaccurate or not recorded -Other information, such as place of birth, was not recorded in the chart
Wiedmeyer, Lofters, & Rashid, (2012) [44]	Retrospective cohort (2004 to 2008)	Toronto, Ontario	To examine if refugee women at a community health centre were appropriately screened for cervical cancer, and what characteristics affect whether they were screened	-Chart review of all patient records from the community health centre from 2004–2008 (sampling not necessary) - Database search of all registered clients of Access Alliance Multicultural Health and Community Services meeting criteria within the specified timeline	357	-Lack of power due to sample size -Low external validity -Did not analyze provider effects (such as male or female physician, demeanor)
Jarvis, Munoz, Graves, et al., (2011) [46]	Retrospective cohort (2004 to 2007)	Montréal, Québec	To assess prenatal and perinatal health outcomes among uninsured pregnant women in Montréal	-Random sampling to obtain insured cohort and convenience sampling to obtain uninsured cohort -Database and chart record audit during specified time period	143	-Difficult population to study as uninsured are often undocumented -Study is not representative of uninsured women with no prenatal care (low external validity) -One of the family health centres provided financial assistance to women -Difficult to collect sociodemographic information -Confounders may have been missed

*IFHP* Interim Federal Health Program, *OHIP* Ontario Health Insurance Plan

The definition of uninsured varied, but generally was described as individuals living in Canada who were not eligible for public health insurance either through the IFHP or provincial health insurance coverage. One study included not having private insurance as part of the definition of uninsured [23]. Another identified uninsured individuals as those who were billed through the Compassionate Care Program; a program offering free primary care services to uninsured patients [41]. Those who were entitled to health insurance but lacked documentation were also included as uninsured [47, 48]. The studies altogether covered a wide scope of uninsured populations (Table 1).

Studies investigated refugees or refused refugee claimants [22, 42–46], asylum seekers awaiting their refugee claim or those who were denied [22, 42, 43, 46], new permanent residents or immigrants [42, 43, 45, 48], visitors [22, 41, 46], undocumented [43, 45, 46], those with no legal or permanent migrant status [22, 41], foreign students and their dependents [22, 46], temporary foreign workers and their dependents [22], those awaiting sponsorship [46], those who were self-paying and reported a permanent address [48], landed immigrants in the three-month waiting period [41], those with a lost or expired health card [41], those who entered the country through non-regular means [43], and those who did not provide a reason for their uninsured status [41]. Two studies provided less specific definitions for participants such as authorized and unauthorized migrants [23] and insured and uninsured [47]. The number of uninsured individuals studied ranged from 52 uninsured individuals sampled from one hospital in Toronto, Ontario [41] to 140,730 uninsured individuals captured in the National Ambulatory Care Reporting System over a span of nine years [48].

In terms of sex, age, and ethnicity, most studies did not report detailed demographics of the uninsured population. Five studies focused on uninsured females as the objective was to assess perinatal outcomes, service utilization, and cervical cancer screening [42, 44, 46, 47, 49]. One study focused on children exclusively [45] and one study explored differences in outcomes by age [48]. No studies clearly analyzed health outcomes or health services use among medically uninsured older adults over the age of 65. Only one study provided data on ethnicities of uninsured individuals, although it was used for descriptive purposes only [43]. The study reported that the highest number of uninsured individuals were from a Caribbean background (40%) followed by South Asian (10%). An additional three studies, two of which reported on the same population, reported region of origin or birth [19, 20, 28].

### Cost

Our search did not produce any results on health care costs among the uninsured population in Canada. There were no quantitative studies that captured the out-of-pocket cost that uninsured individuals may have to pay while accessing physician or hospital services. Two studies discussed cost, however both were excluded at the data extraction stage as they examined the financial effect of the 2012 cuts to the IFHP on refugee claimants and therefore did not meet our inclusion criteria [50, 51]. Moreover, no studies investigated the cost implications of having private insurance in the uninsured populations. This suggests a need for quantitative research on the financial impact of medical uninsurance.

### Health service utilization

Nine included studies investigated health services use among uninsured individuals [22, 41–48]. Many of these studies concluded that utilization of health care services such as emergency room and physician visits, and hospital admissions were impeded by a lack of coverage (Table 2). Two studies showed that those without insurance were more likely to be triaged into a severe category upon arrival to the hospital compared to those who were insured, with Rousseau et al. (2013) suggesting this may be attributed in part to a delay in seeking care [31, 34]. Ridde et al. (2020) reported that the reasons for unmet health care needs among uninsured individuals included not having enough money to pay fees (81%), fear of being overcharged (73%), potential negative impact of health consultation on migration status (22%), and fear of rejection by the hospital (7%) [22]. They also mentioned that among those who used health care services, the majority accessed private pharmacies (60%) and community organized health services (43%), while hospitals were used by fewer individuals (14%) [22].

**Table 2:** Health service utilization reported in uninsured population

Author & year of publication	Service accessed	Main results
Ridde, Aho, Ndao, et al., (2020) [22]	-Private pharmacies -Community organized health clinics -Walk-in clinics -Dental clinics -Hospitals	-Unreported health care needs were reported by 69% of uninsured migrants in comparison to 26% of recent immigrants and 16% of citizens with insurance. Unmet health care needs were greatest among temporary workers and their descendants (73%) -The association between unmet health care needs and migrant status was not statistically significant -Reasons for unmet health care needs included not having enough money to pay fees (81%), fear of being overcharged (73%), potential negative impact of health consultation on migration status (22%), and fear of rejection by hospital (7%) -Almost one fifth (19%) of all participants reported not knowing where to access health care -Among those who used health care services, they accessed private pharmacies (60%), community organized health services (43%), walk-in-clinics (21%), dental clinics (16%), and hospitals (14%) -Services such as osteopathy, chiropractic, and physiotherapy were used by less than 3%
Darling, Bennett, Burton, et al., (2019) [47]	-Antenatal services -Intrapartum services -Postpartum services	-Uninsured migrant women compared to insured women: -Attended fewer antenatal appointments (mean 9.9 visits vs. 11.6 visits) -Had more antenatal home visits (mean 1.9 visits vs. 0.6 visits) -Were less likely to attend a prenatal visit in the first trimester (66.3% vs. 92.8%) -Presented later to midwifery care (18.4 weeks gestation vs. 12.7 weeks gestation) -Were less likely to attend prenatal class (33.2% vs. 65.2% for primiparous participants and 2.9% vs. 5.7% for multiparous participants) -Had shorter hospital stays when they gave birth at the hospital (median 2 h vs. 3 h) -Had more intrapartum consultations for fetal well-being and meconium while consultations for labour dystocia, oxytocin augmentation, and epidural were less common -Received more postpartum home visits (mean 3.7 visits vs. 3.2 visits) - Planned for home birth more (33.9% planned home birth and 28.7% gave birth at home whereas 19.6% insured planned home birth and 16.6% gave birth at home) -Were less likely to have at least one postpartum consultation with a physician (5.5% vs. 6.8%) -Were more likely to have a registered midwife (64.7% vs. 60.2%) -Had lower transfer of care in labour (6.1% vs. 23.8%) -Had lower newborn intensive care unit admissions (8.7% vs. 9.2%) -Had lower newborn metabolic screening (90.9% vs. 92%) -Had lower severity in care level. Level 1 hospitals were low-need and level 3 hospitals were high-need. (Level 1: 12.4% vs. 12.2%; Level 2: 77.3% vs. 74.5%; Level 3: 10.6% vs. 13.4%) -The proportion of uninsured clients varied across the province, with midwifery clinics in the South West, Central, and Toronto Central Local Health Integration Networks caring for the highest percentage of uninsured clients
Hynie, Ardern, & Robertson (2016) [48]	-Emergency room -Hospital	-The percentage of visits of uninsured increased from 0.23% in 2002/3 to 0.44% in 2010/11 -Within Ontario, the proportion of visits by the uninsured to the emergency room ranged from 0.07% in Erie St. Clair to 0.66% in Toronto -Visit disposition differed by insurance status as those without insurance were less likely to be admitted (10.2% insured vs. 9.4% uninsured), more likely to leave without treatment (3.1% insured vs. 5.4% uninsured), and more likely to have died on arrival or in the emergency room (2.8% insured vs. 3.7% uninsured) -Emergency room visits related to ambulatory care sensitive conditions were more common among the insured than uninsured (4.55% vs. 3.18%) -A larger proportion of ambulatory care sensitive condition visits were accounted for by children (≤ 16 years), and youth (17–24 years) in the uninsured group -Insured and uninsured were equally likely to be triaged into one of the severe categories if they arrived with ambulatory care sensitive conditions -At hospital presentation, 15.6% of uninsured and 11.2% of insured individuals were triaged into one of the more severe categories (resuscitation or emergent) -Compared to insured individuals (Odds Ratio (OR(1.00, referent), those who were uninsured were 43% more likely (OR 1.43; 95% Confidence Interval (CI): 1.39–1.46) to be classified as severe, even after adjustment for age and gender
Bunn, Fleming, Rzeznikiewiz, et al., (2013) [41]	-Prenatal care -Pediatric care	-No significant difference between participants with Ontario Health Insurance Plan vs. participants utilizing the Compassionate Care Program in the proportion of patients seeking prenatal or routine pediatric care -6% and 16% of insured and uninsured used prenatal care, respectively (p value (p) = 0.184) -18% and 11% of insured and uninsured used routine pediatric care, respectively (*p* = 0.344)
Gagnon, Merry, & Haase (2013) [42]	-Newborn intensive care unit admission -Hospital	-Significant differences in newborn intensive care unit admission were found among refugees (26.7%), asylum seekers (15.6%) and immigrants (7.0%), where *p* = 0.073
Rousseau, Laurin-Lamothe, Rummens, et al., (2013) [45]	-Emergency room	-1.3% of uninsured children compared to 0.3% of federally insured refugee children had the highest level of emergency (level 1) (chi-squared statistic (x^2^) = 15,290.01; *p* < 0.001) and 11.5% of uninsured children vs. 8.5% of federally insured refugee children had the second highest level of emergency (level 2) (x^2^ = 89,055.93; *p* < 0.001). For levels 3–5, there were no significant differences between groups -In hospitals 2 (Montreal) (t = 4.81; *p* < 0.001) and 3 (Toronto) (t statistic (t) = 6.83; *p* < 0.001) the mean emergency rating at triage for uninsured immigrant and refugee claimant children was significantly higher (less urgent) than the mean emergency rating of the overall hospital populations -In hospital 1 (Montreal), the refugee claimant and uninsured children status mean emergency rating was comparable with the overall hospital population mean emergency rating (t = -1.62; *p* = 0.105) -Hospitalization of refugee claimants was more frequent in hospital 1 (25.1%) in Montreal compared to hospital 2 (2.5%) in Montreal and hospital 3 (9.2%) in Toronto [ *p* < 0.001] -In hospital 2 (Montreal), 82.6% of children were prescribed medication, compared with 55.7% in hospital 3 (Toronto) and 34.3% in hospital 1 (Montreal) ( *p* < 0.001) -In both hospital 1 and hospital 3, the overall number of children leaving with another follow-up plan documented in the file was approximately 20%; in contrast, it was 2.0% in hospital 2, where 10.6% of children also left before ever seeing a doctor
Wilson-Mitchell & Rummens, (2013) [43]	-Number of prenatal visits -Provider type -Length of stay in hospital for mother and baby	-Uninsured mothers had shorter hospital stays than insured mothers (1.7 days vs. 2.4 days) ( t = − 6.110) -No significant difference for baby length of stay between insured and uninsured mothers -36.6% of uninsured saw a registered midwife vs. 4.0% of insured -55.4% of uninsured saw an obstetrician vs. 94.1% of insured -Number of prenatal visits for the uninsured group was significantly lower than the insured group (mean 6.04 vs. 8.70; t = − 6.173) -6.5% of uninsured women received no prenatal care whereas 100% of insured women received prenatal care. An equal proportion saw a general practitioner (1.8%) -Using guidelines by the Society of Obstetricians and Gynaecologists of Canada, more than half (53.7%) of the uninsured women received inadequate prenatal care in comparison to one-in-five (19.6%) insured women
Wiedmeyer, Lofters, & Rashid, (2012) [44]	-Cervical cancer screening (Pap test)	-75% of insured women had a Pap test; 95% of refugee and uninsured women had a Pap test -Univariate analysis: uninsured women were significantly more likely to have Pap tests compared to insured women (OR 6.65; *p* < 0.0001) -Multivariate analysis: when controlling for confounders (language, region of origin, year of arrival, pregnancy, and age), there was no significant differences in receiving a Pap test between the insured women and uninsured women (adjusted hazard ratio 1.312; 95% CI: 0.922–2.058) -In the cox proportional hazard model, insurance status was significantly associated with time to first Pap test (adjusted hazard ratio = 1.715; 95% CI: 1.156–2.545). Although uninsured patients were more likely to get a Pap test at any point in time in this model, after adjustment for all main regions of origin, rather than simply using the stratification of European versus non-European, this result became non-significant (adjusted hazard ratio 1.312; 95% CI: 0.922 to 2.058) -English speakers had a higher likelihood of having a Pap test early compared to non-English speakers (adjusted hazard ratio 0.625 95%; CI: 0.462–0.854)
Jarvis, Munoz, Graves, et al., (2011) [46]	-Routine prenatal services (including blood tests, obstetric ultrasound, cervical swab for sexually transmitted infections, Pap tests and early genetic screening) -Postpartum services -Visits with health care professionals	-Uninsured women had fewer initial screening blood tests conducted (93.7% vs. 100%; *p* = 0.045), ultrasound screenings (82.5% vs. 98.4%; *p* = 0.003), cervical swabs (69.8% vs. 85.2%; *p* = 0.04), Pap tests (38.1% vs. 75.4%; *p* < 0.001), genetic screenings (12.7% vs. 44.3%; *p* < 0.001), lower mean total number of prenatal visits (6.6 visits; 3.4 SD vs. 10.7 visits; 3.0 SD; *p* = 0.05), and less physical examinations (6.6% vs. 10.7%; not statistically significant) -Gestational age at first visit for uninsured women was 25.6 weeks vs. 12 weeks for insured women (*p* < 0.001) -Using the Prenatal Care Utilization Index, the uninsured vs. insured experienced inadequate (61.9% vs. 11.7%), intermediate (12.7% vs. 13.3%), adequate (20.6% vs. 55%), and adequate care with prenatal care utilization (4.8% vs. 20%). The difference between the groups was significant (x^2^ = 36.3; *p* = 0.001) -In terms of adequacy of received services, the uninsured vs. insured experienced inadequate (6.3% vs. 1.7%), intermediate (33.3% vs. 15%), adequate (44.4% vs. 58.3%), and adequate care plus (15.9% vs. 25%). The difference was statistically significant (x^2^ = 8.3; *p* = 0.04) -Uninsured pregnant women presented for initial care 13.6 weeks later than insured women (25.6 weeks vs. 12.0 weeks; *p* < 0.001)

*OR*  odds ratio, *p* *p*-value, *x*^*2*^ chi-squared statistic, *t* t-statistic

Most studies focused on healthcare services targeted towards females including prenatal and maternal care, midwifery, obstetrician access, and cervical screening. Regarding access to prenatal services, three studies reported reduced utilization among uninsured women compared to insured [43, 46, 47]. These services included prenatal visits, obstetrician services, and prenatal screening such as blood tests, cervical swabs, Pap tests, ultrasounds, and genetic screenings. Wilson-Mitchell & Rummens (2013) found that 6.5% of uninsured women received no antenatal care, whereas 100% of the insured received care [43]. Among uninsured women that received care, only 55% saw an obstetrician, compared to 94% of insured women. Uninsured women were significantly more likely to have sought the services of a midwife (36.0% vs. 4.0%), to have presented at a later gestational age (18.4 weeks vs. 12.7 weeks) and to have delivered their baby at home compared to the insured (28.7% vs. 16.6%) [29, 33]. Those delivering in the hospital had shorter stays compared to insured mothers [43, 47]. Contrary to these findings, Wiedmeyer, Lofters, & Rashid (2012) reported that uninsured women were more likely to have had a Pap test compared to insured women (95.0% vs. 75.0%, odds ratio (OR) = 6.65; *p* < 0.0001) [44]. However, when the regression was adjusted for variables such as age and English-speaking ability, the strength of the association was not significant (OR = 2.71; 95% confidence interval (CI) = 0.80–9.26). Notably, the sample size for this retrospective chart review was small and the results may not be generalizable since it was from a health care centre that provided care specifically to uninsured individuals and had an established support system for refugees and new immigrants. Jarvis et al. (2011) studied the amount of prenatal and perinatal care accessed by uninsured pregnant women at two primary care centres, one of which provided services free of charge [46]. Uninsured women had fewer prenatal visits than insured women and were more likely to have received inadequate care, which may be due to having started care later in their pregnancy. Jarvis et al. (2011) also conducted a site comparison which revealed uninsured women attended more appointments and were more likely to have received adequate care at the centre providing services free of charge [46]. The results indicate that providing prenatal and perinatal care services free of charge may increase utilization among uninsured women.

The quality assessment of these studies identified limitations with selected study designs (Table 3). As the majority were cross-sectional and retrospective studies, effects of temporality need to be taken into consideration when interpreting results. The reviewed data primarily originated from the emergency room and community health care centres which may not accurately represent the uninsured population in Canada. Since these centres were not randomly selected, extrapolation of the results may be misleading. Another validity concern in most of the studies is that the reported results were not adjusted for known confounders. Rather than running rigorous analytical regressions adjusting for covariates, many studies solely conducted descriptive analysis.

**Table 3:** Risk of bias

Author & year of publication	Bias due to confounding	Bias in selection of participants into the study	Bias in classification of interventions	Bias due to deviations from intended interventions	Bias due to missing data	Bias in measurement of outcomes	Bias in selection of the reported result
Cloos, Ndao, Aho, et al., (2020) [23] Overall bias: Low	Low Multivariate regression conducted and controlled for many variables	Low Non-probability sampling at a clinic was employed to recruit uninsured which may have introduced selection bias. Bias reduced by also recruiting from venue-based sampling and social media	Low Clear definition for uninsured	Low Study conducted around the time of Interim Federal Health Program (IFHP) reinstatement which was not addressed. Individuals who were unaware that they were eligible for the IFHP or who had benefited from it in the past but had not been able to extend or renew it were also included. Excluded those with private insurance or IFHP	Low Little missing data-did not exceed 5% for most variables and for the three variables that had 12%, they were excluded	Low Questionnaire was developed using the Trajectory Model and was validated in migrant and general populations. Questionnaire was available in multiple languages. May have limited interviewer bias given that research assistants knew that they were interviewing uninsured individuals. The outcome was self-reported	Low Reported all analysis conducted
Ridde, Aho, Ndao, et al., (2020) [22] Overall bias: Low	Low Multivariate regression conducted and controlled for many variables	Low Non-probability sampling at a clinic was employed to recruit uninsured which may have introduced selection bias. Bias reduced by also recruiting from venue-based sampling and social media	Low Clear definition of uninsured	Low Study conducted around the time of IFHP reinstatement which was not addressed. Individuals who were unaware that they were eligible for the IFHP or who had benefited from it in the past but had not been able to extend or renew it were also included. Excluded those with private insurance or IFHP	Low Little missing data-did not exceed 5% for most variables and for the three variables that had 12%, they were excluded	Low Questionnaire was developed using the Trajectory Model and was validated in migrant and general populations. Questionnaire was available in multiple languages. May have limited interviewer bias given that research assistants knew that they were interviewing uninsured individuals. The outcome was self-reported	Low Reported all analysis conducted
Darling, Bennett, Burton, et al., (2019) [47] Overall bias: Low	Moderate Potential for confounders that were not measured and not controlled for in the analysis (little information regarding sociodemographic factors, missing parity or maternal age)	Low Population-based cohort of all midwifery clients who gave birth between 2012 and 2015	Moderate BORN-Ontario registry does not detail why participants were uninsured. They defined intervention group to be those that did not have Ontario Health Insurance Plan (OHIP) and comparator group to be those with OHIP	Low Excluded those whose insurance status was unclear	Low BORN-Ontario had high completion of data	Low Study used retrospective BORN-Ontario administrative data which had validation checks, but retrospective chart review is problematic due to inaccuracy and inconsistency in recording	Low Reported all analysis conducted and numbers of individuals excluded. The numbers in the text are not the same as those reported in the tables. Confidence intervals are not reported
Hynie, Ardern, & Robertson (2016) [48] Overall bias: Moderate	Moderate Potential for confounders that were not measured and not controlled for (age and sex were adjusted for but socioeconomic factors were not)	Moderate Administrative dataset used. Looked at main diagnoses for insured and uninsured clients in a 10% subsample-how they selected them not specified	Low Analysis of 9 consecutive years of data reduced the impact of temporality. Uninsured definition as those who were self-paying. Reason for self-paying not addressed	Moderate People would only pay out-of-pocket or pay through insurance. Unclear whether people had private insurance and would get reimbursed later	Moderate Not addressed	Moderate International Classification of Diseases (ICD) codes may have differed across the 9 years as not all hospitals adopted it in 2002. The software was assessed and said to be valid but there was under-reporting of multiple problems and lower agreement of main problem for those with multiple problems	Low Reported all analysis conducted. Did not report p-values and confidence intervals for all analysis
Bunn, Fleming, Rzeznikiewiz, et al., (2013) [41] Overall bias: Moderate	Serious Study was descriptive-no modelling conducted. No covariates controlled for	Moderate Small sample size. Had a 1:1 case to control ratio. Did not describe randomization process for selecting controls -Case and controls were different in median income	Low A lot of the uninsured population did not provide reason for being uninsured so unclear why they billed Compassionate Care Program	Moderate Unclear whether all those who billed through Compassionate Care Program had no form of insurance	Moderate Uninsured had greater amount of missing charts and had missing information on income	Moderate Researchers were unsure if every participant was screened for all of the diagnoses investigated. Retrospective chart reviews are problematic due to inaccuracy and inconsistency in recording	Low Reported all analysis conducted
Gagnon, Merry, & Haase (2013) [42] Overall bias: Moderate	Low Several confounding variables were controlled for. Used regression modelling	Low Sampled from 12 hospitals. Selected individuals from set categories and selected controls with closest date and date of birth to a case	Moderate Used pre-set definitions to identify refugees, asylum seekers, and immigrants. Did not describe health insurance variable	Moderate Study was before cuts made to IFHP. Whether uninsured had federal insurance or private insurance was unclear	Moderate Reduced model presented when data was missing. Did not investigate missing data further	Low Obtained data prospectively through interviews as well as medical records. Questionnaires were available in multiple languages. Data verification protocol was utilized	Moderate Did not present full regression results
Rousseau, Laurin-Lamothe, Rummens, et al., (2013) [45] Overall bias: Moderate	Moderate Sociodemographic information was not reported. Potential for confounders that were not measured and not controlled for	Moderate The hospitals involved in the study were not randomly selected. The hospitals differed from one another in their population and samples from these hospitals were chosen differently	Low Uninsured were those who didn’t have OHIP or Régie de l'assurance maladie du Québec (RAMQ) as recorded by medical files	Moderate Did not exclude those who may have private insurance or IFHP	Moderate Not addressed	Moderate Hospitals differed in their record-keeping. One hospital in particular had a migrant outpatient and so could be biased in reporting. Retrospective chart reviews can be problematic due to inaccuracy and inconsistency	Low Reported all analysis conducted
Wilson-Mitchell & Rummens, (2013) [43] Overall bias: Moderate	Serious Potential for confounders that were not measured and not controlled for	Serious The uninsured charts were not sampled randomly Sampling methods were not further explained. Did not match cases and controls on relevant factors so unsure whether they were similar in everything except for insurance status	Low Used hospital payment codes to identify insurance status	Low Uninsured included those without provincial coverage. Excluded homeless women, those with IFHP, private insurance, or insurance from another province. Study predates changes to IFHP	Moderate Not addressed	Moderate Retrospective chart reviews are problematic due to inaccuracy and inconsistency in recording	Low Reported all analysis conducted
Wiedmeyer, Lofters, & Rashid, (2012) [44] Overall bias: Moderate	Low Confounders included but missing a few such as marital status	Moderate 63 charts identified for review were unable to be retrieved for this study, which could have led to selection bias	Moderate Uninsured and insured definitions not provided	Moderate Study predated changes to IFHP. Not clear whether there was deviation	Moderate Not addressed	Moderate Retrospective chart reviews are problematic due to inaccuracy and inconsistency in recording	Moderate Authors performed further analysis after seeing unexpected results, not specified a priori
Jarvis, Munoz, Graves, et al., (2011) [46] Overall bias: Serious	Moderate Many confounding variables not included	Moderate Convenience sampling utilized-locations were affiliated with each other but differed in access for uninsured. Control sample was selected randomly from same hospitals (did not specify the randomization process), but demographic variables were not compared between cases and controls to know if the groups were comparable	Moderate Used medical records from initial presentation to identify insurance status. The status could have changed over time	Moderate One of the sites had funding for refugee referrals and provided financial assistance for tests and visits to uninsured. Excluded those with IFHP or private insurance	Low Did not compare those who were lost to follow-up. Did not discuss missing data from medical records	Moderate Retrospective chart reviews are problematic due to inaccuracy and inconsistency in recording	Low Reported all analysis conducted

*IFHP* Interim Federal Health Program, *OHIP* Ontario Health Insurance Plan, *ICD* International Classification of Disease, *RAMQ* Régie de l'assurance maladie du Québec

### Health outcomes

Table 4 outlines nine studies that discussed health outcomes in the context of Medicare uninsurance among the Canadian migrant population [22, 23, 41–43, 45–48]. Comparing results from across studies, the most common outcome reported among uninsured individuals was poor mental health [22, 23, 41, 45, 48]. Hynie et al. (2016) reported a prevalence of mental and behavioural issues at 10.5% vs. 3.5% in the uninsured and insured, respectively [48]. Likewise, Rousseau et al. (2013) mentioned that compared to refugee children, uninsured children were more likely to access the emergency department for depression, post-traumatic stress disorder, suicidal thoughts, and substance use [45]. Focusing on the uninsured population, Cloos et al. (2020) reported that 26% of their sample had mental distress [23].

**Table 4:** Health outcomes reported in uninsured population

First author & year of publication	Health outcome	Main results
Cloos, Ndao, Aho, et al., (2020) [23]	-Self-perceived health (bad, fair, good, very good, excellent) -Psychological distress measured by the Kessler 6 scale -Health issues in the last year	-527 (68.9%) uninsured migrants reported unmet health needs -348 (44.6%) of all migrants perceived their health as negative -192 (26.3%) of migrants reported mental distress -652 (83.5%) of migrants had a health problem within the past 12 months
Ridde, Aho, Ndao, et al., (2020) [22]	-Self-perceived health -Psychological distress measured by the Kessler 6 scale -Unmet healthcare needs	-36.9% of the migrants reported receiving a diagnosis by a healthcare professional. Among these individuals, the most frequently reported diagnosis included cardiovascular and circulatory disease (34%), mental health issues (14%), endocrine system complications (26%), and musculoskeletal system complications (11%)
Darling, Bennett, Burton, et al., (2019) [47]	-Gestational age at birth -Mode of birth -Type of labour -Reasons for induction -Fetal health surveillance -Pharmacological pain management -Intrapartum complications -Preterm birth weight -Rate of small for gestational age -Exclusive breastfeeding at 6 months	-Uninsured participants had higher rates of spontaneous labour than insured (82.1% vs. 77.3%) and spontaneous vaginal birth (81.1% vs. 78.1%) -Uninsured had lower rates of induction of labour than insured (13.5% vs. 17%), electronic fetal monitoring (12.6% vs. 14.6%), assisted vaginal birth (4.7% vs. 5.8%) and Cesarean Sects. (13.9% vs. 15%) -Uninsured more likely to not use pain medication during labour than insured (46.7% vs. 37.1%) -Uninsured compared to insured had postpartum hemorrhage (3.4% vs. 2.9%), preterm birth (5% vs. 4.4%) and gave birth to small for-gestational-age babies (2.1% vs. 1.7%) -At 6 weeks, exclusive breastfeeding was lower among the uninsured (75.1% vs. 78.1%) -Uninsured compared to insured had gestational age at birth < 29 weeks (0.5% vs. 0.3%), 29–33 weeks (0.7% vs. 0.7%), 34–36 weeks (3.9% vs. 3.4%), 37–38 weeks (18.9% vs. 17.8%), 39–41 weeks (74.7% vs. 76.2%) and > 41 weeks (1.4% vs. 1.6%)
Hynie, Ardern, & Robertson (2016) [48]	-Severity of diagnosis: hypertension, chronic obstructive pulmonary disease, coronary heart failure, diabetes, angina -Mental/behavioural health -Obstetrics outcomes, death, injury	-Uninsured compared to insured in Ontario: -Diagnosis for ambulatory care sensitive conditions was higher: 4.55% vs. 3.18% -Mental health diagnosis were three times higher: 10.5% vs. 3.5% -Obstetric complications were higher: 5.6% vs. 2.7% -More likely to die on arrival or in the emergency room: 3.7% vs. 2.8% -The three most common diagnoses among the insured were: injury (24.5%), other clinical/lab (18.4%), and respiratory (11.2%) -The three most common diagnoses among uninsured were injury (28.4%), other clinical/lab (17.0%), and mental health (10.5%)
Bunn, Fleming, Rzeznikiewiz, et al., (2013) [41]	-Specific diagnosis including hypertension, type 2 diabetes, HIV, tuberculosis, substance addiction, or mental health disorder	-Hypertension (14% insured vs. 16% uninsured; p-value(p) = 0.831) -Type 2 diabetes (6% insured vs. 11% uninsured; *p* = 0.470) -HIV (4% insured vs. 24% uninsured; *p* = 0.004) -Tuberculosis (6% insured vs. 13% uninsured; *p* = 0.300) -Substance addiction (6% insured vs. 4% uninsured; *p* > 0.99) -Mental health disorders (14% insured vs. 16% uninsured; *p* = 0.831)
Gagnon, Merry, & Haase (2013) [42]	-Emergency Cesarean delivery -Planned Cesarean or vaginal delivery	-Among migrant women, no health insurance coverage compared to health insurance coverage (provincial, IFHP, or private) was associated with higher risk for emergency Cesarean delivery (Odds Ratio (OR), 2.8; 95% Confidence Interval (CI): 1.2–6.3) -Compared to immigrants, being an asylum seeker (OR = 0.3; 95% CI: 0.2–0.6) or refugee (OR = 0.5; 95% CI: 0.2–1.0) was protective
Rousseau, Laurin-Lamothe, Rummens, et al., (2013) [43]	-Triage level of emergency care -Medical and social problems reported -Treatment and follow-up	Compared to refugee claimant children, uninsured children presented more often for: -Musculoskeletal injuries or lacerations (12.1% refugee vs. 20.7% uninsured; *p* < 0.001) -Depression (0.4% refugee vs. 3% uninsured; *p* < 0.001) -Post-traumatic stress disorder (0% refugee vs. 0.4% uninsured; *p* < 0.001) -Suicidal thoughts (0.8% refugee vs. 2.3% uninsured; *p* = 0.008) -Substance abuse (0.2% refugee vs. 0.9% uninsured; *p* = 0.048) Compared to uninsured children, refugee claimant children were more frequently diagnosed with: -Respiratory virus infection (30.2% refugee vs. 23.4% uninsured; *p* = 0.001) -Abdominal pain (4% refugee vs. 2.3% uninsured; *p* = 0.035) -Sickle cell anaemia (3.5% refugee vs. 1.4% uninsured; *p* = 0.005) -Appendicitis (1.3% refugee vs. 0.2% uninsured; *p* = 0.009) Non-significant differences were: -Gastroenteric virus (9.6% refugee vs. 7.7% uninsured) -Bacterial infection (6.1% refugee vs. 6.3% uninsured) -Eczema/rash (3.4% refugee vs. 2.4% uninsured) -Asthma (1.8% refugee vs. 1.6% uninsured) -Behavioural problems such as opposition and relational problems (1.6% refugees vs. 2.5% uninsured) -Pervasive developmental disorder (1.5% refugee vs. 0.8% uninsured) -Negligence (0.1% refugee vs. 0.1% uninsured)
Wilson-Mitchell & Rummens, (2013) [43]	-Cesarean section rate -Maternal and neonatal complications -Low birth weight -Small for gestational age -Preterm birth -Newborn intensive care unit admission -Exclusive breastfeeding at discharge -Intrapartum care	-There were no significant differences between uninsured and insured women for low birth weight, preterm birth, maternal complications, intrapartum care and exclusive breastfeeding -Newborns of uninsured mothers had a significantly higher incidence (9.7% vs. 4.3% with chi-squared statistic (x^2^) = 5.174) of neonatal resuscitation. The difference in newborn intensive care unit admission was not significant (15.16% insured; CI: 10.94–19.39 vs. 14.37% uninsured; CI: 9.16–19.58) -Cesarean sections occurred more in the insured group than the uninsured (35.6% vs. 26.3%; x^2^ = 4.292) but uninsured women had a significantly higher rate of Cesarean sections due to abnormal fetal heart rate (35% vs. 21.7%; x^2^ = 5.405) whereas most common reason for C-section in insured women was labor dystocia
Jarvis, Munoz, Graves, et al., (2011) [46]	-Perinatal outcomes such as gestational age and birth weight of insured and uninsured participants’ baby -Route of delivery (vaginal birth, Cesarean section)	-Uninsured migrant women had lower gestational age at birth than their insured counterparts (39.0 weeks vs. 39.2 weeks) and gave birth to babies with lower birth weight than their insured counterparts (3,379 g vs. 3,387 g rams) -Vaginal birth was higher in uninsured women (71.4% vs. 69.5%; not statistically significant) and Cesarean sections were lower (28.6% vs. 30.5%; not statistically significant). In terms of delivery interventions, epidural use was lower among uninsured (71.4% vs. 73.3%; not statistically significant) and induction was higher (25.4% vs. 20%; not statistically significant)

*OR* odds ratio, *p*  *p*-value, *x*^*2*^ chi-squared statistic

Other studies [45, 48] showed an association between uninsured status and adverse health outcomes [48]. For example, Hynie et al. (2016) found that obstetrical complications, resuscitation, and death upon arrival to the emergency room occurred more among uninsured compared to insured individuals at 5.6% vs. 2.7%, 15.6% vs. 11.2%, and 3.7% vs. 2.8%, respectively [48]. Uninsured compared to insured migrant children and youth in Ontario also showed higher diagnosis and prevalence for Ambulatory Care Sensitive Conditions at 4.6% vs. 3.2%. Bunn et al. (2013) also found greater HIV (human immunodeficiency virus) diagnosis among uninsured patients in comparison to insured patients (24% vs. 4%) [41].

Apart from mental health outcomes, there was no clear relationship between one particular health outcome and insurance status. Hynie et al. (2016) reported that while injury, poor mental health, and obstetric outcomes were more frequently reported among the uninsured population, skin disease, eye disease, and respiratory conditions were more frequently reported among the insured [48]. Likewise, Wilson-Mitchell et al. (2013) showed that while gestational diabetes was higher in those uninsured than insured, the opposite was true for hypertension [43]. Both studies however did not test for statistical significance. Rousseau et al. (2013) tested for significance and showed that refugee claimants were significantly more frequently diagnosed with respiratory virus infections, abdominal pain, and appendicitis while uninsured children reported musculoskeletal injuries or lacerations and mental health conditions such as depression, posttraumatic stress disorder, or suicidal thoughts more often (χ2 = 6.97; *p* = 0.008) [45]. Similar to other studies, Bunn et al. (2013) showed no statistically significant difference between the insured and uninsured groups with respect to many outcomes studied including hypertension, type 2 diabetes, and tuberculosis [41].

Four studies investigated the relationship between Medicare uninsurance and maternal health outcomes including gestational age at birth, type of labour, fetal health, intrapartum and postpartum complications, and preterm birth weight among women [42, 43, 46, 47]. In comparison to their insured counterparts, Darling et al. (2019) found uninsured pregnant women had higher rates of postpartum hemorrhage (3.4% vs. 2.9%), preterm birth (5.0% vs. 4.4%), and babies who were small for gestational age (2.1% vs. 1.7%) [47]. Both Darling et al. (2019) and Jarvis et al. (2011) also reported lower Caesarean section rate (26.3% vs. 35.6%; 13.9% vs. 15.0%) among uninsured compared to insured pregnant women [46, 47]. The most common reason for a Caesarean section among insured and uninsured women was labor dystocia and abnormal fetal heart rate respectively [43]. Gagnon, Merry & Haase (2013) also reported the absence of health insurance as a risk factor for higher emergency Caesarean delivery among migrant women (OR, 2.8; 95% CI: 1.2–6.3) [28]. Uninsured women had babies with lower birth weight than insured women, but the difference was not found to be statistically significant [43, 46]. Also, Wilson-Mitchell & Rummens (2013) reported no significant difference between low birth rate, preterm birth, breastfeeding rates, overall maternal complications, and intrapartum medical interventions among insured and uninsured women [43]. Research by Wilson-Mitchell & Rummens (2013) [29] and Darling et al. (2019) [47] was limited by using a retrospective cohort design. Furthermore, the authors did not address nor adjust for confounding factors. The chart review technique utilized by Wilson-Mitchell & Rummens (2013) also had challenges with accuracy and consistency [43]. Other studies accounted for confounders where possible, however, they were limited by lack of randomization, small sample size, and potential selection bias.

Three studies suggested that the determining factor behind poor health outcomes among uninsured patients was the three-month waiting period to receive health care coverage [41, 45, 48]. However, neither of these studies focused exclusively on the three-month waiting period. These studies received moderate overall bias ratings using the ROBINS-I tool. While Hynie et al. (2016) accounted for age and sex, other confounding variables such as measures of socioeconomic status were not addressed [48]. Rousseau et al. (2013) did not account for confounding variables, nor did the authors address any sources of missing data [45]. Finally, Bunn et al. (2013) had a small sample size which may affect generalizability of their results [41].

### Overall quality assessment

The overall risk of bias for the included studies ranged from low to serious (Table 3). Three studies had a low risk of bias rating [22, 23, 47], six studies had a moderate rating [41–45, 48], and one study was rated as serious [46]. Studies with a low risk of bias rating typically controlled for confounders, addressed missing data, and reported all analysis conducted. Reasoning for a moderate rating included small sample sizes, confounders not addressed, and concerns with selection bias due to the sampling methods used. Given that most studies used retrospective databases or medical chart reviews, variables including socio-demographic information were often unavailable and therefore could not be adjusted for. There may have also been inaccuracy and inconsistency in reporting in studies using a retrospective chart review. There were also concerns with selection bias in some of the studies, especially in those sampling from non-randomly selected hospitals or clinics. One study received a serious risk of bias rating as it did not consider important confounders, used medical records that may have changed over time, did not account for missing data, did not compare those who were lost to follow up, and utilized a retrospective chart review [46].

Two included cross sectional studies utilized the same population of migrants in Montreal, Quebec [22, 23]. The studies differed in their purposes as Cloos et al. (2020) focused on the association between precarious migration status and self-perceived health [23] whereas Ridde et al. (2020) examined unmet health care needs and its associated factors among uninsured migrants [22]. Cloos et al. (2020) reported on health outcomes [23] while Ridde et al. (2020) reported on both health outcomes and health service utilization among migrants [22] and thus we felt it necessary to include both studies in the review.

## Discussion

This systematic review examined multiple databases and grey literature sources to identify studies exploring the health outcomes, utilization, and cost consequences of Medicare uninsurance among the migrant population in Canada. The results showed that the medically uninsured population is very understudied in Canada. Other Canadian reviews conducted with narrower inclusion criteria have reported a similar number of included studies [3, 6, 42]. Reasons for limited studies on medically uninsured populations include ethical barriers to study this population and limitations of existing data collection methods [52, 53]. Gagnon et al. (2021) who conducted a narrative scoping review on immigration status as a determinant of health, which we see as a complementary study to ours, showed that studies in this area are primarily qualitative in nature [16]. Our search also highlighted that literature is limited by the definition of medically uninsured. Although the inclusion criteria of the review ensured only studies researching provincially medically uninsured populations were captured, the search proved it challenging to make comparisons across all studies because of the differences in how researchers defined uninsured and insured.

Our review demonstrated a gap in Canadian quantitative literature on the medically uninsured population’s out-of-pocket costs when accessing medically necessary services. There are also no economic studies that evaluate the financial impact of medically uninsured populations on the health care system. This restricts policymakers from understanding the scope of the problem. This gap should be addressed given that case studies demonstrate cost as an impeding factor to care. Caulford & D’Andrade (2012) published a case study of an 18-year old female who was told her case was not an emergency when she visited the emergency room for her sickle cell crisis [17]. While waiting in triage, she fainted and was hospitalized for three days, costing her $5,000.

Our results revealed that health services use was low when there was a lack of health insurance. For instance, some uninsured migrant women did not receive any prenatal care whatsoever [43]. This is of particular concern as prenatal care is widely regarded as effective and cost-saving with research suggesting that for every $1 US spent on prenatal care, there are $2 of savings [54]. Consistent with our findings, a scoping review by Magalhaes et al. (2010) found that undocumented migrant workers in Canada had reduced health service utilization due to limited access to health care stemming from fear of deportation, unaffordability of services, lack of knowledge of the health care system, and social isolation [3]. A study conducted by Allen et al. (2017) also reported systemic-level barriers and discrimination as a major factor behind low health care service use [55]. Notably, one study in our review examined a community health centre in Toronto and found the opposite effect [44]. Uninsured refugee women were more likely to receive cervical cancer screening than insured women. While rates of cervical cancer screening are low in the migrant population, this could be because the community clinic model removed barriers to care by providing interpreters, offering settlement services, and providing care to the uninsured free of cost [44]. This health care model could be useful for decision-makers as an example of how to accommodate the needs of this population and provide appropriate care The same trend was observed with the use of midwives when there was a cost associated with visiting physician or hospital services that they could not afford [43]. Physicians or personal social networks may direct women towards midwifery and community health centres that are providing services at little to no cost.

This review did not provide high quality evidence on health outcomes among uninsured populations as half of the studies used descriptive analysis without measures of associations and tests of significance and without adjusting for confounding factors. Our results indicated that uninsured women were at a greater risk of poor obstetric outcomes such as preterm births, emergency Caesarean sections, and postpartum hemorrhages [42, 43] which may be attributed to the absence of health care insurance. Moreover, new permanent residents reported mental distress, poor self-perceived health, and unmet health care needs which suggests the three-month waiting period may contribute to adverse effects on health and wellbeing [45]. This finding is consistent with previous research [6, 56]. A scoping review conducted on new permanent residents in Canada found the mandated three-month waiting period for health insurance created a barrier to accessing necessary care which negatively affected health outcomes [6].

Our review also revealed a greater prevalence of mental health issues such as depression and suicidal thoughts among uninsured migrants compared to their insured counterparts. Uninsured migrants often face poverty, systemic racism, trauma, lack of sufficient support systems, and added pressures when transitioning to a new environment [23, 57]. This exacerbates existing mental health issues as they cannot access services to address their needs [23]. Similar results have been reported in other countries with high numbers of migrants with precarious status [58, 59]. The growing body of qualitative Canadian studies have also reported similar findings [60]. Interviews conducted by Goel et al. (2013) indicated that participants experienced emotional hardship during the three month waiting period including fear, affecting their mental health [32].

Lastly, conducting quality assessment showed the limitations of the current literature including small sample sizes, lack of administrative data, and lack of rigorous analytical methods. The latter could have been because of the limitations of the minimal data that is available on uninsured migrants. In fact, prior to COVID-19, many organizations did not collect data on ethnicity and immigrant status. It is known from the literature that racism and ethnicity/race influence health outcomes and health care use of individuals [61, 62]. As such, racism and/or ethnicity/race may in fact interact with immigrant status to influence outcomes among the uninsured.

This review highlighted that it is impossible to know the current state of health outcomes and health care use among the medically uninsured at a population level if data is not available. It is unclear whether appropriate data is not being collected at the organizational level or whether organizations are not reporting that data. It points to a need to evaluate community and province-level data sources and assess what type of data is being collected and what is missing. At the same time, it becomes important to have a discussion on how best to collect data from uninsured individuals who are often very vulnerable and given that the process can create greater barriers between uninsured groups and the health care system.

There is also a need to create linked administrative datasets that show the services uninsured and insured individuals use over their lifetime. Linked databases will also help address the problem of missing data, especially that from the community. Given the challenges of quantitative data, this review also highlights the need to conduct multi-methods studies that include qualitative research. That being said, strengths of quantitative research should also be mentioned. This review presented the size of the health and health care problems that affect uninsured migrants. It also highlighted statistical differences between insured and uninsured groups for certain conditions and services.

### Strengths and limitations

Our review provides a comprehensive analysis of the health outcomes and health services use among medically uninsured migrants in Canada. We utilized the Cochrane ROBINS-1 tool which allowed us to assess the quality of existing studies on the limitations of current literature. Our review also has a few limitations. First, we only included studies with sufficient quantitative data to extract. There are many qualitative studies focused on the uninsured population in Canada that could have provided interesting insights. Qualitative findings may have allowed us to gain a deeper understanding of reported health outcomes and trends in health care service use seen quantitatively in non-insured migrants. It could also further contextualize our findings, help fill the missing quantitative data gaps, especially with respect to cost data, and help policymakers understand the problem in greater depth. Additionally, the heterogeneity of included studies presents a limitation when interpreting results. Each study had a different definition of ‘uninsured’, and while some definitions shared common themes, some studies could have inadvertently included individuals who were not migrants, as chart reviews did not always include the reason for being uninsured or migratory status. Also, given the limited number of studies included, we could not do any sub-group analyses by uninsured group across studies (i.e., undocumented vs. permanent residents within three-month waiting period). We were also unable to make causal claims given the nature of the studies we included. Lastly, the review was restricted to the limits of the questions posed and search terms included a priori. Although we were interested in including and extracting French papers, we did not find any relevant studies in French. This may be because our search terms were in English.

### Implications and future research

Our findings can be used to inform policy decisions regarding the provision of health insurance for migrants. Given the observed poor health outcomes, policymakers should consider how providing preventive care to migrants could be beneficial for population health and overall costs. Providing preventive care could help avoid expensive hospitalizations and improve health outcomes among the uninsured. Removing the three-month wait period for health coverage for new permanent residents should be considered as it could improve health by providing more timely care and therefore reduce costs for the government. Further, policymakers may consider increasing funding for community centres as they are commonly utilized by the medically uninsured [44]. Community health centres may also consider expanding their staff to include professionals specializing in mental health, pregnancy, and chronic illnesses as disparities in these areas were frequently reported in our review. An integrated medical system that is universal for all may also be created rather than having a two-tier system in which the uninsured have to rely on community organizations and face barriers in accessing care.

Future research may consider analyzing specific barriers that migrants face when accessing health insurance. Cost as a barrier to seeking care was not discussed in detail. This likely has a large impact on service usage as the cost of care in Canada is expensive for those who do not have medical insurance. Moreover, our literature search revealed a lack of clear data on the older migrant population in Canada. The elderly often experience chronic health conditions and multiple comorbidities. It is important to investigate service utilization in this population as they require additional support from the health care system such as long-term care. Future research may also consider focusing on areas such as specific mental health issues, chronic diseases, and therapy services to better understand the burden of medical uninsurance among migrants. Lastly, included studies were primarily conducted in Ontario and Quebec which reduces generalizability of results to other provinces. British Columbia is home to a very large migrant population and yet there are minimal quantitative studies conducted in this province, and only one study included in our review [7, 42]. As previously mentioned, this could be due to limited relevant data collection and data sources on the health and health care use of medically uninsured migrants. Researching health outcomes and health care services use of medically uninsured populations using linked datasets that include more data from the community as well as sociodemographic data is greatly needed.

## Conclusion

This review builds on existing evidence by demonstrating how lack of insurance influences health outcomes, reduces health service utilization, and inhibits access to necessary care. There is a need to provide better access to affordable health care services for the medically uninsured population. We hope our findings can be used to inform policy decisions with the overall goal of improving inequities in health outcomes and service usage for migrants residing in Canada.

## Supplementary Information

**Additional file 1:** Appendices.

## Data Availability

All data generated or analyzed during this study are included in this published article [and its supplementary information file].

## Abbreviations

IFHP: Interim Federal Health Program
ROBINS-I: Risk of Bias in Non-randomized Studies of Interventions
PRISMA: Preferred Reporting Items for Systematic reviews and Meta-Analyses
HIV: Human Immunodeficiency Virus
OR: Odds Ratio
CI: Confidence Interval
P: P-value
X2: Chi-squared statistic
T: T-statistic
OHIP: Ontario Health Insurance Plan
ICD: International Classification of Disease
RAMQ: Régie de l'assurance maladie du Québec
