# Supplementary material for: Health outcomes, health services utilization, and costs consequences of medicare uninsurance among migrants in Canada: a systematic review
Source: BMC Health Serv Res. 2023 May 3;23:427. doi: 10.1186/s12913-023-09417-4 (PMC10154752; doi:10.1186/s12913-023-09417-4)
Supplement: Supplementary file 1 — Additional file 1: Appendices. [file 12913_2023_9417_MOESM1_ESM.docx]

**Health Outcomes, Health Services Utilization, and Costs Consequences of Medicare Uninsurance among Migrants in Canada: A Systematic Review**

**Supplementary Document**

Sophiya Garasia1,2 *, Valerie Bishop 1, Stephanie Clayton 1, Genevieve Pinnington 1 , Chika Arinze 1, 3, Ezza Jalil 1

1. Department of Health Research Methods, Evidence, and Impact, McMaster University, Hamilton, Ontario, Canada

2. Centre for Health Economics and Policy Analysis, McMaster University, Hamilton, Ontario, Canada

3. Department of Oncology, McMaster University, Hamilton, Ontario, Canada

*Corresponding Author

Name: Sophiya Garasia

Mailing Address: 1280 Main Street West, Hamilton, Ontario, Canada, L8S 4K1

Phone Number: 905-525-9140 ext. 24658

Email Address: [garasis@mcmaster.ca](mailto:garasis@mcmaster.ca)

**Appendix I: Search Strategy and Search Terms**

*Area*

- Canada
- Ontario
- Quebec
- Alberta
- Manitoba
- Saskatchewan Prince Edward Island
- New Brunswick
- Nova Scotia
- Newfoundland
- British Columbia

*Population*

- Non-citizen
- Non-status
- Immigrant and emigrants
- Migrant
- Refugee
- Newcomers
- New permanent resident
- Temporary foreign workers
- Migrant workers
- Asylum seekers
- Uninsured
- Undocumented
- Undocumented immigrant
- Immigration status
- Uninsured residents
- Refugee claimants

*Intervention*

- Health insurance
- Medicare
- Canadian Medicare
- Canada Health Act
- OHIP 90 day waiting period
- OHIP 3 month waiting period
- Universal health coverage
- Universal health care coverage
- Provincial health card
- Interim Federal Health Program
- OHIP
- Alberta health care insurance plan
- Medical services plan
- Insured benefits branch of Manitoba
- New Brunswick medicare
- Newfoundland and Labrador medical care plan
- NWT health care plan
- Nova Scotia health
- Nunavut health care plan
- Ontario health insurance plan
- Health PEI
- Government of Saskatchewan
- Quebec health insurance plan
- RAMQ
- Yukon health care insurance plan
- YHCIP

*Health*

- Health
- Health outcomes
- Health status
- Health care need
- Health care delivery
- Delivery of health care
- Reproductive health
- Health care disparities
- Women’s health
- Health equity
- Social determinants of health
- Family health
- Sexual health
- Child health
- Injury rates
- Mental health
- Maternal health
- Occupational health
- Perinatal health
- Pregnancy outcomes
- Adolescent health
- Health care quality

*Utilization*

- Mental health care
- Health care service
- Emergency care
- Prenatal care
- Community care
- Primary health care
- Health literacy
- Health care access
- Emergency health service
- Emergency medical services
- Health seeking behaviours
- Primary care
- Preventative care
- Acute care
- Health access
- Barriers
- Community care use
- Community health centre
- Health care delivery
- Hospitalization
- Physician services
- Hospital services
- Surgery
- Laboratory services
- Inpatient services
- Outpatient services
- Postnatal care
- Obstetrical care

*Cost*

- Cost
- Health care costs
- Health economics
- Payment
- Hospital costs
- Provincial health care costs
- Provincial health expenditure
- Health expenditure
- Out-of-pocket costs
- Out-of-pocket expenditure
- Private health insurance
- Medical bills

| **Date** | | March 9, 2021 |
| --- | --- | --- |
| **Database** | | OVID MEDLINE Epub Ahead of Print, In-Process & Other Non-Indexed Citations, Ovid MEDLINE(R) Daily and Ovid MEDLINE(R) 1946 to Present |
| **Institution** | | McMaster University Library |
| **Search Strategy:** | 1 | (non-citizen or non-status or immigrant* or migrant* or refugee* or newcomer* or new permanent resident or temporary foreign worker or migrant worker* or asylum seeker or uninsured or undocumented or undocumented immigrant or immigration status or uninsured resident or refugee claimant*).mp. |
|  | 2 | (canada or ontario or quebec or alberta or manitoba or saskatchewan or prince edward island or new brunswick or nova scotia or newfoundland or british columbia).mp. |
|  | 3 | 1 and 2 |
|  | 4 | (health insurance or Medicare or Canadian Medicare or Canada Health Act or OHIP 90 day waiting period or OHIP 3 month waiting period or universal health coverage or universal health care coverage or provincial health card or Interim Federal Health Program or OHIP or alberta health care insurance plan or medical services plan or insured benefits branch of manitoba or new brunswick medicare or (newfoundland and labrador medical care plan) or nwt health care plan or nova scotia health or nunavut health care plan or ontario health insurance plan or health pei or government of saskatchewan or quebec health insurance plan or RAMQ or yukon health care insurance plan or YHCIP).mp. |
|  | 5 | (health or health outcomes or health status or health care need or health care delivery or delivery of healthcare or reproductive health or healthcare disparities or women's health or health equity or social determinants of health or family health or sexual health or child health or injury rates or mental health or mental health outcomes or maternal health or occupational health or perinatal health or pregnancy outcomes or adolescent health or health care quality).mp. |
|  | 6 | (cost* or health care costs or health economics or payment or hospital costs or provincial health care costs or provincial health expenditure or health expenditure or out-of-pocket costs or out-of-pocket expenditure or private health insurance or medical bill*).mp. |
|  | 7 | (health care service* or emergency care or mental health care or prenatal care or community care or primary health care or health literacy or health care access or emergency health service* or emergency medical service* or health seeking behaviour* or preventative care or acute care or barriers or community care usage or health care delivery or hospitalization or physician services or hospital services or surgery or Laboratory services or inpatient services or outpatient services or postnatal care or obstetrical care).mp. |
|  | 8 | 5 or 6 or 7 |
|  | 9 | 4 and 8 |
|  | 10 | 3 and 9 |
| **Hits** | | 109 |
| **Limits** | | None |

| **Date** | | March 9, 2021 |
| --- | --- | --- |
| **Database** | | **Embase**1996 to 2021 March 02 |
| **Institution** | | McMaster University Library |
| **Search Strategy:** | 1 | (non-citizen or non-status or immigrant* or migrant* or refugee* or newcomer* or new permanent resident or temporary foreign worker or migrant worker* or asylum seeker or uninsured or undocumented or undocumented immigrant or immigration status or uninsured resident or refugee claimant*).mp. |
|  | 2 | (canada or ontario or quebec or alberta or manitoba or saskatchewan or prince edward island or new brunswick or nova scotia or newfoundland or british columbia).mp. |
|  | 3 | 1 and 2 |
|  | 4 | (health insurance or Medicare or Canadian Medicare or Canada Health Act or OHIP 90 day waiting period or OHIP 3 month waiting period or universal health coverage or universal health care coverage or provincial health card or Interim Federal Health Program or OHIP or alberta health care insurance plan or medical services plan or insured benefits branch of manitoba or new brunswick medicare or (newfoundland and labrador medical care plan) or nwt health care plan or nova scotia health or nunavut health care plan or ontario health insurance plan or health pei or government of saskatchewan or quebec health insurance plan or RAMQ or yukon health care insurance plan or YHCIP).mp. |
|  | 5 | (health or health outcomes or health status or health care need or health care delivery or delivery of healthcare or reproductive health or healthcare disparities or women's health or health equity or social determinants of health or family health or sexual health or child health or injury rates or mental health or mental health outcomes or maternal health or occupational health or perinatal health or pregnancy outcomes or adolescent health or health care quality).mp. |
|  | 6 | (cost* or health care costs or health economics or payment or hospital costs or provincial health care costs or provincial health expenditure or health expenditure or out-of-pocket costs or out-of-pocket expenditure or private health insurance or medical bill*).mp. |
|  | 7 | (health care service* or emergency care or mental health care or prenatal care or community care or primary health care or health literacy or health care access or emergency health service* or emergency medical service* or health seeking behaviour* or preventative care or acute care or barriers or community care usage or health care delivery or hospitalization or physician services or hospital services or surgery or Laboratory services or inpatient services or outpatient services or postnatal care or obstetrical care).mp. |
|  | 8 | 5 or 6 or 7 |
|  | 9 | 4 and 8 |
|  | 10 | 3 and 9 |
| **Hits** | | 156 |
| **Limits** | | None |

| **Date** | | March 9, 2021 |
| --- | --- | --- |
| **Database** | | **Global Health**1973 to 2021 Week 08 |
| **Institution** | | McMaster University Library |
| **Search Strategy:** | 1 | (non-citizen or non-status or immigrant* or migrant* or refugee* or newcomer* or new permanent resident or temporary foreign worker or migrant worker* or asylum seeker or uninsured or undocumented or undocumented immigrant or immigration status or uninsured resident or refugee claimant*).mp. |
|  | 2 | (canada or ontario or quebec or alberta or manitoba or saskatchewan or prince edward island or new brunswick or nova scotia or newfoundland or british columbia).mp. |
|  | 3 | 1 and 2 |
|  | 4 | (health insurance or Medicare or Canadian Medicare or Canada Health Act or OHIP 90 day waiting period or OHIP 3 month waiting period or universal health coverage or universal health care coverage or provincial health card or Interim Federal Health Program or OHIP or alberta health care insurance plan or medical services plan or insured benefits branch of manitoba or new brunswick medicare or (newfoundland and labrador medical care plan) or nwt health care plan or nova scotia health or nunavut health care plan or ontario health insurance plan or health pei or government of saskatchewan or quebec health insurance plan or RAMQ or yukon health care insurance plan or YHCIP).mp. |
|  | 5 | (health or health outcomes or health status or health care need or health care delivery or delivery of healthcare or reproductive health or healthcare disparities or women's health or health equity or social determinants of health or family health or sexual health or child health or injury rates or mental health or mental health outcomes or maternal health or occupational health or perinatal health or pregnancy outcomes or adolescent health or health care quality).mp. |
|  | 6 | (cost* or health care costs or health economics or payment or hospital costs or provincial health care costs or provincial health expenditure or health expenditure or out-of-pocket costs or out-of-pocket expenditure or private health insurance or medical bill*).mp. |
|  | 7 | (health care service* or emergency care or mental health care or prenatal care or community care or primary health care or health literacy or health care access or emergency health service* or emergency medical service* or health seeking behaviour* or preventative care or acute care or barriers or community care usage or health care delivery or hospitalization or physician services or hospital services or surgery or Laboratory services or inpatient services or outpatient services or postnatal care or obstetrical care).mp. |
|  | 8 | 5 or 6 or 7 |
|  | 9 | 4 and 8 |
|  | 10 | 3 and 9 |
| **Hits** | | 36 |
| **Limits** | | None |

| **Date** | | March 9, 2021 |
| --- | --- | --- |
| **Database** | | **EconLit with Full Text** |
| **Institution** | | McMaster University Library |
| **Search Strategy:** | 1 | canada OR ontario OR quebec OR alberta OR manitoba OR saskatchewan OR (prince edward island) OR (new brunswick) OR (nova scotia) OR newfoundland OR (british columbia) |
|  | 2 | non-citizen OR non-status OR immigrant* OR migrant* OR refugee* OR newcomer* OR (new permanent resident) OR (temporary foreign worker) OR (migrant worker*) OR (asylum seeker) OR uninsured OR undocumented OR (undocumented immigrant) OR (immigration status) OR (uninsured resident) OR (refugee claimant*) |
|  | 3 | 1 and 2 |
|  | 4 | (health insurance) OR Medicare OR (Canadian Medicare) OR (Canada Health Act) OR (OHIP 90 day waiting period) OR (OHIP 3 month waiting period) OR (universal health coverage) OR (universal health care coverage) OR (provincial health card) OR (Interim Federal Health Program) OR OHIP OR (alberta health care insurance plan) OR (medical services plan) OR (insured benefits branch of manitoba) OR (new brunswick medicare) OR (newfoundland and labrador medical care plan) OR (nwt health care plan) OR (nova scotia health) OR (nunavut health care plan) OR (ontario health insurance plan) OR (health pei) OR (government of saskatchewan) OR (quebec health insurance plan) OR RAMQ OR (yukon health care insurance plan) OR YHCIP |
|  | 5 | health OR (health outcomes) OR (health status) OR (health care need) OR (health care delivery) OR (delivery of healthcare) OR (reproductive health) OR (healthcare disparities) OR (women's health) OR (health equity) OR (social determinants of health) OR (family health) OR (sexual health) OR (child health) OR (injury rates) OR (mental health) OR (mental health outcomes) OR (maternal health) OR (occupational health) OR (perinatal health) OR (pregnancy outcomes) OR (adolescent health) OR (health care quality) |
|  | 6 | cost* OR (health care costs) OR (health economics) OR payment OR (hospital costs) OR (provincial health care costs) OR (provincial health expenditure) OR (health expenditure) OR (out-of-pocket costs) OR (out-of-pocket expenditure) OR (private health insurance) OR (medical bill*) |
|  | 7 | (health care service*) OR (emergency care) OR (mental health care) OR (prenatal care) OR (community care) OR (primary health care) OR (health literacy) OR (health care access) OR (emergency health service*) OR (emergency medical service*) OR (health seeking behaviour*) OR (preventative care) OR (acute care) OR (barriers) OR (community care usage) OR (health care delivery) OR hospitalization OR (physician services) OR (hospital services) OR surgery OR (Laboratory services) OR (inpatient services) OR (outpatient services) OR (postnatal care) OR (obstetrical care) |
|  | 8 | ((health care service*) OR (emergency care) OR (mental health care) OR (prenatal care) OR (community care) OR (primary health care) OR (health literacy) OR (health care access) OR (emergency health service*) OR (emergency medical service*) OR (health seeking behaviour*) OR (preventative care) OR (acute care) OR (barriers) OR (community care usage) OR (health care delivery) OR hospitalization OR (physician services) OR (hospital services) OR surgery OR (Laboratory services) OR (inpatient services) OR (outpatient services) OR (postnatal care) OR (obstetrical care)) AND (S5 OR S6 OR S7) |
|  | 9 | (((health care service*) OR (emergency care) OR (mental health care) OR (prenatal care) OR (community care) OR (primary health care) OR (health literacy) OR (health care access) OR (emergency health service*) OR (emergency medical service*) OR (health seeking behaviour*) OR (preventative care) OR (acute care) OR (barriers) OR (community care usage) OR (health care delivery) OR hospitalization OR (physician services) OR (hospital services) OR surgery OR (Laboratory services) OR (inpatient services) OR (outpatient services) OR (postnatal care) OR (obstetrical care)) AND (S5 OR S6 OR S7)) AND (S4 AND S8) |
|  | 10 | ((((health care service*) OR (emergency care) OR (mental health care) OR (prenatal care) OR (community care) OR (primary health care) OR (health literacy) OR (health care access) OR (emergency health service*) OR (emergency medical service*) OR (health seeking behaviour*) OR (preventative care) OR (acute care) OR (barriers) OR (community care usage) OR (health care delivery) OR hospitalization OR (physician services) OR (hospital services) OR surgery OR (Laboratory services) OR (inpatient services) OR (outpatient services) OR (postnatal care) OR (obstetrical care)) AND (S5 OR S6 OR S7)) AND (S4 AND S8)) AND (S3 AND S9) |
| **Hits** | | 8 |
| **Limits/Expanders** | | Apply equivalent subjects |

**Appendix II: List of Excluded Studies**

**Irrelevant Study Design (27)**

Vanthuyne, K., Meloni, F., Ruiz-Casares, M., Rousseau, C., & Ricard-Guay, A. (2013). Health workers perceptions of access to care for children and pregnant women with precarious immigration status: Health as a right or a privilege? *Social Science & Medicine,* *93*, 78-85. doi:10.1016/j.socscimed.2013.06.008

Winn, A., Hetherington, E., & Tough, S. (2018). Caring for pregnant refugee women in a turbulent policy landscape: Perspectives of health care professionals in Calgary, Alberta. *International Journal for Equity in Health,* *17*(1). doi:10.1186/s12939-018-0801-5

Stanbrook, M. B. (2014). Canada owes refugees adequate health coverage. *Canadian Medical Association Journal,* *186*(2), 91-91. doi:10.1503/cmaj.131861

Caulford, P. (2006). Providing health care to medically uninsured immigrants and refugees. *Canadian Medical Association Journal,* *174*(9), 1253-1254. doi:10.1503/cmaj.051206

Asanin, J., & Wilson, K. (2008). “I spent nine years looking for a doctor”: Exploring access to health care among immigrants in Mississauga, Ontario, Canada. *Social Science & Medicine,* *66*(6), 1271-1283. doi:10.1016/j.socscimed.2007.11.043

Berry, R. S. (2004). Physician Support for Covering and Caring for the Uninsured. *Annals of Internal Medicine,* *141*(3), 244. doi:10.7326/0003-4819-141-3-200408030-00023

Munro, K., Jarvis, C., Kong, L. Y., D'Souza, V., & Graves, L. (2013). Perspectives of family physicians on the care of uninsured pregnant women. *Journal of Obstetrics and Gynaecology Canada*, *35*(7), 599-605. <https://dx.doi.org/10.1016/S1701-2163>

Kent, H. (2000). BC doctor seeks class-action suit over payment for uninsured patients. *Canadian Medical Association Journal, 163 (9) 1184;*

Marks, J. H. (2013). The Undocumented Unwell.*Hastings Center Report, 43(1), 10-11.* doi:10.1002/hast.124

Goel, R., & Beder, M. (2011). Welcome to Canada … but don’t get sick. *Canadian Medical Association Journal,* *184*(1). doi:10.1503/cmaj.111095

Hennebry, J. L., & Williams, G. (2015). Making vulnerability visible: Medical repatriation and Canada’s migrant agricultural workers. *Canadian Medical Association Journal,* *187*(6), 391-392. doi:10.1503/cmaj.141189

Narushima, M., & Sanchez, A. L. (2014). Employers´ paradoxical views about temporary foreign migrant workers´ health: A qualitative study in rural farms in Southern Ontario. *International Journal for Equity in Health,* *13*(1). doi:10.1186/s12939-014-0065-7

Lynas, k. (2011). Quebec Pharmacists and Federal Immigration Department Reach Understanding on Dispute over Drug Coverage for Refugees. *Canadian Pharmacists Journal / Revue Des Pharmaciens Du Canada,* *144*(2), 54-54. doi:10.3821/1913-701x-144.2.54

Antonipillai, V., Abelson, J., Wahoush, O., Baumann, A., & Schwartz, L. (2020). Policy Agenda-Setting and Causal Stories: Examining How Organized Interests redefined the Problem of Refugee Health Policy in Canada. *Healthcare Policy | Politiques De Santé,15*(3), 116-131. doi:10.12927/hcpol.2020.26126

Rink, N., Muttalib, F., Morantz, G., Chase, L., Cleveland, J., Rousseau, C., & Li, P. (2017). The gap between coverage and care—what can Canadian paediatricians do about access to health services for refugee claimant children? *Paediatrics & Child Health,* *22*(8), 430-437. doi:10.1093/pch/pxx115

Edmonds, J., & Flahault, A. (2021). Refugees in Canada during the First Wave of the COVID-19 Pandemic. *International Journal of Environmental Research and Public Health,* *18*(3), 947. doi:10.3390/ijerph18030947

Paterson, J. M. (2004). Informed consent for uninsured services: A primary care perspective on the new childhood vaccines. *Canadian Medical Association Journal,* *171*(8), 877-879. doi:10.1503/cmaj.1031738

Goel, R., Bloch, G., & Caulford, P. (2013). Waiting for care: Effects of Ontario’s 3-month waiting period for OHIP on landed immigrants. *Canadian Family Physician*, *59*(6), e269-e275.

Gagnon, A., Bouris, S., Merry, L. (2013). Developmental delays in children of international migrants: Family profiles. *European Journal of Epidemiology, 28(1), S166.*

Nielsen, L. S., Goldstein, Z., Leung, D., Lee, C., & Buick, C. (2019). A Scoping Review of Undocumented Immigrants and Palliative Care: Implications for the Canadian Context.*Journal of Immigrant and Minority Health, 21(6), 1394-1405.* doi:10.1007/s10903-019-00882-w

López, L., DesRoches, C. M., Vogeli, C., Grant, R. W., Iezzoni, L. I., & Campbell, E. G. (2013). Characteristics of primary care safety-net providers and their quality improvement attitudes and activities: results of a national survey of physician professionalism. *American Journal of Medical Quality*, *28*(2), 151-159.

Kumar, B., Caulford, P., & Lee, R. (2002). Volunteer clinic for the uninsured. *Canadian Family Physician*, *48*, 869.

Caulford, P., & D’Andrade, J. (2012). Health care for Canada’s medically uninsured immigrants and refugees: Whose problem is it?. *Canadian Family Physician*, *58*(7), 725-727.

Guruge, S., Sidani, S., Illesinghe, V., Younes, R., Bukhari, H., Altenberg, J., . . . Fredericks, S. (2021). Healthcare needs and health service utilization by Syrian refugee women in Toronto. doi:10.32920/14638857.v1

Miedema, B., Hamilton, R., & Easley, J. (2008). Climbing the walls: structural barriers to accessing primary care for refugee newcomers in Canada. *Canadian Family Physician*, *54*(3), 335-336.

Evans, A., Caudarella, A., Ratnapalan, S., & Chan, K. (2014). The cost and impact of the interim federal health program cuts on child refugees in Canada. *PloS one*, *9*(5), e96902.

Abdelaal, M., Blake, C., & Lau, J. (2021). Challenges of Providing Palliative and End-of-Life Care to Refugee Claimants in Canada: A Case Report. *Journal of Palliative Medicine*, *24*(4), 635-638.

**Irrelevant Patient Population (26)**

Schoen, C., & Doty, M. M. (2004). Inequities in access to medical care in five countries: Findings from the 2001 Commonwealth Fund International Health Policy Survey. *Health Policy,* *67*(3), 309-322. doi:10.1016/j.healthpol.2003.09.006

Zhang, E., Razik, F., & Ratnapalan, S. (2018). MP05: Injuries in refugee children presenting to a paediatric emergency department. *Canadian Journal of Emergency Medicine*, *20*(S1), S41-S42.

Paszat, L., Sutradhar, R., Liu, Y., Baxter, N. N., Tinmouth, J., & Rabeneck, L. (2017). Risk of colorectal cancer among immigrants to Ontario, Canada. *BMC Gastroenterology,* *17*(1). doi:10.1186/s12876-017-0642-5

Siddiqi, A. A., Wang, S., Quinn, K., Nguyen, Q. C., & Christy, A. D. (2016). Racial Disparities in Access to Care Under Conditions of Universal Coverage. *American Journal of Preventive Medicine,* *50*(2), 220-225. doi:10.1016/j.amepre.2014.08.004

Lofters, A. K., Moineddin, R., Hwang, S. W., & Glazier, R. H. (2011). Predictors of low cervical cancer screening among immigrant women in Ontario, Canada. *BMC Womens Health,* *11*(1). doi:10.1186/1472-6874-11-20

Lofters, A. K., Moineddin, R., Hwang, S. W., & Glazier, R. H. (2010). Low Rates of Cervical Cancer Screening Among Urban Immigrants. *Medical Care,48*(7), 611-618. doi:10.1097/mlr.0b013e3181d6886f

Shen, S.C., Lofters, A., Tinmouth, J., Paszat, L., Rabeneck, L., & Glazier, R. H. (2018). Predictors of non-adherence to colorectal cancer screening among immigrants to Ontario, Canada: A population-based study. *Preventive Medicine,* *111*, 180-189. doi:10.1016/j.ypmed.2018.03.002

Kennedy, J., & Morgan, S. (2006). Health Care Access in Three Nations: Canada, Insured America, and Uninsured America. *International Journal of Health Services,* *36*(4), 697-717. doi:10.2190/ec30-kp22-ra84-ral4

Fazli, G. S., Moineddin, R., Bierman, A. S., & Booth, G. L. (2020). Ethnic variation in the conversion of prediabetes to diabetes among immigrant populations relative to Canadian-born residents: A population-based cohort study. *BMJ Open Diabetes Research & Care,* *8*(1). doi:10.1136/bmjdrc-2019-000907

Fuchs, V. R., & Hahn, J. S. (1990). How does Canada do it? A comparison of expenditures for physicians' services in the United States and Canada. *New England Journal of Medicine*, *323*(13), 884-890.

Landry, M. D., Deber, R. B., Jaglal, S., Laporte, A., Holyoke, P., Devitt, R., & Cott, C. (2006). Assessing the consequences of delisting publicly funded community-based physical therapy on self-reported health in Ontario, Canada: A prospective cohort study. *International Journal of Rehabilitation Research,29*(4), 303-307. doi:10.1097/mrr.0b013e328010badc

Burchell, A. N., Kendall, C. E., Cheng, S. Y., Lofters, A., Cotterchio, M., Bayoumi, A. M., . . . Loutfy, M. (2018). Cervical cancer screening uptake among HIV-positive women in Ontario, Canada: A population-based retrospective cohort study. *Preventive Medicine,107*, 14-20. doi:10.1016/j.ypmed.2017.11.023

Caring for the Uninsured and Underinsured. (1991). *JAMA*, *266*(15), 2079-2080.

Kennedy, J., & Morgan, S. (2009). Cost-related prescription nonadherence in the United States and Canada: a system-level comparison using the 2007 International Health Policy Survey in Seven Countries. *Clinical therapeutics*, *31*(1), 213-219.

Rotstein, D. L., Marrie, R. A., Tu, K., Schultz, S. E., Fung, K., & Maxwell, C. J. (2020). Health service utilization in immigrants with multiple sclerosis. *Plos One,* *15*(7). doi:10.1371/journal.pone.0234876

Guttmann, A., Manuel, D., Stukel, T. A., Desmeules, M., Cernat, G., & Glazier, R. H. (2008). Immunization Coverage Among Young Children of Urban Immigrant Mothers: Findings from a Universal Health Care System. *Ambulatory Pediatrics,* *8*(3), 205-209. doi:10.1016/j.ambp.2008.01.010

Batista, R., Pottie, K. C., Dahrouge, S., Manuel, D. G., Tanuseputro, P., Mark, A. E., & Ng, E. (2019). Impact of health care reform on enrolment of immigrants in primary care in Ontario, Canada. *Family practice*, *36*(4), 445-451.

Wanigaratne, S., Shakya, Y., Gagnon, A. J., Cole, D. C., Rashid, M., Blake, J., . . . Urquia, M. L. (2018). Refugee maternal and perinatal health in Ontario, Canada: A retrospective population-based study. *BMJ Open,* *8*(4). doi:10.1136/bmjopen-2017-018979

Daw, J. R., Rice, K. E., & Raza, D. (2020). Fees for uninsured services: A cross-sectional survey of Ontario family physicians. *CMAJ Open,* *8*(1). doi:10.9778/cmajo.20190189

Rotstein, D. L., Marrie, R. A., Maxwell, C., Ghandi, S., Schultz, S. E., Fung, K., & Tu, K. (2018). MS Risk in Immigrants in the McDonald Era: A Population-Based Study in Ontario, Canada. *SSRN Electronic Journal*. doi:10.2139/ssrn.3252689

Nugent, Z., Chan, K., Hilliard, R.I., Ford-Jones, E.L., Caulford P.(2011). Children Without Medical Insurance in a Canadian Metropolitan City. *Paediatrics and Child Health*. doi:10.1093/pch/16.suppl_a.42aa

Evans, A., Caudarella, A., Ratnapalan, S., & Chan, K. (2014). The cost and impact of the interim federal health program cuts on child refugees in Canada. *PloS one*, *9*(5), e96902.

Bakewell, F., Addleman, S., Dickinson, G., & Thiruganasambandamoorthy, V. (2018). Use of the emergency department by refugees under the Interim Federal Health Program: A health records review. *Plos one*, *13*(5), e0197282.

Kandasamy, T., Cherniak, R., Shah, R., Yudin, M. H., & Spitzer, R. (2014). Obstetric risks and outcomes of refugee women at a single centre in Toronto. *Journal of Obstetrics and Gynaecology Canada*, *36*(4), 296-302.

Oda, A., Tuck, A., Agic, B., Hynie, M., Roche, B., & McKenzie, K. (2017). Health care needs and use of health care services among newly arrived Syrian refugees: a cross-sectional study. *CMAJ open*, *5*(2), E354.

Lebrun, L. A. (2012). Effects of length of stay and language proficiency on health care experiences among immigrants in Canada and the United States. *Social science & medicine*, *74*(7), 1062-1072.

**Irrelevant Intervention (10)**

Ronald, L. A., Campbell, J. R., Balshaw, R. F., Roth, D. Z., Romanowski, K., Marra, F., . . . Johnston, J. C. (2016). Predicting tuberculosis risk in the foreign-born population of British Columbia, Canada: Study protocol for a retrospective population-based cohort study. *BMJ Open,* *6*(11). doi:10.1136/bmjopen-2016-013488

Hyman, I., Shakya, Y., Jembere, N., Gucciardi, E., & Vissandjée, B. (2017). Provider-and patient-related determinants of diabetes self-management among recent immigrants: Implications for systemic change. *Canadian Family Physician*, *63*(2), e137-e144.

Lum, I. D., Swartz, R. H., & Kwan, M. Y. (2016). Accessibility and use of primary healthcare for immigrants living in the Niagara Region. *Social Science & Medicine,* *156*, 73-79. doi:10.1016/j.socscimed.2016.03.024

David, P., Robert, E., Wong, A., & Sheehan, N. (2020). The relational dimensions of pharmaceutical care: Experience from caring for HIV-infected asylum seekers in Montréal. *Research in Social and Administrative Pharmacy,* *16*(6), 800-804. doi:10.1016/j.sapharm.2019.09.002

Greenaway, C., Cnossen, S., Rossi, C., Schwartzman, K., Holcroft, C., Klein, M. (2011).

The burden of chronic hepatitis B in immigrants in Quebec, Canada: A population based study. *American Journal of Tropical Medicine and Hygiene*, 85(6),394

Arya, A., Brahmania, M., Kowgier, M., Shah, H., Cerocchi, O., Wong, D., ... & Janssen, H. (2015). Clinical Outcomes of Patients with Private vs. Public Drug Coverage for Treatment of Chronic Hepatitis B: 1556. *Hepatology*, *62*.

Dewa, C. S., Hoch, J. S., & Steele, L. (2005). Prescription drug benefits and Canadas uninsured. *International Journal of Law and Psychiatry,* *28*(5), 496-513. doi:10.1016/j.ijlp.2005.08.003

Newbold, K. B., & Danforth, J. (2003). Health status and Canadas immigrant population. *Social Science & Medicine,57*(10), 1981-1995. doi:10.1016/s0277-9536(03)00064-9

Ray, J. G., Vermeulen, M. J., Schull, M. J., Singh, G., Shah, R., & Redelmeier, D. A. (2007). Results of the Recent Immigrant Pregnancy and Perinatal Long-term Evaluation Study (RIPPLES). *Canadian Medical Association Journal,* *176*(10), 1419-1426. doi:10.1503/cmaj.061680

Soon, J., Norman, W. V., Sheila Dunn, M. D., Jennifer Hulme, M. D., & Edith Guilbert, M. D. (2012). Opportunities for optimizing the role of pharmacists in family planning. *Canadian Pharmacists Journal*, *145*(4), S8.

**Irrelevant Outcomes (6)**

Ghahari, S., Burnett, S., & Alexander, L. (2020). Development and pilot testing of a health education program to improve immigrants’ access to Canadian health services. *BMC Health Services Research,20*(1). doi:10.1186/s12913-020-05180-y

Rotstein, D., Maxwell, C., Tu, K., Schultz, S., Fung, K., & Marrie, R. (2020). Risk of Mortality in Immigrants with Multiple Sclerosis in Ontario, Canada. *Neuroepidemiology,* *54*(Suppl. 2), 148-156. doi:10.1159/000506161

Krajewski, S. A., Hameed, S. M., Smink, D. S., & Rogers, S. O. (2009). Access to emergency operative care: A comparative study between the Canadian and American health care systems. *Surgery,* *146*(2), 300-307. doi:10.1016/j.surg.2009.04.005

Gohmann, S. F. (2010). A comparison of health care in Canada and the United States: the case of Pap smears. *Medical care*, 1036-1040.

Reinhardt, U. E. (1987). Resource Allocation in Health Care: The Allocation of Lifestyles to Providers. *The Milbank Quarterly,* *65*(2), 153. doi:10.2307/3350018

Silva, D. S., Cook, V. J., Johnston, J. C., & Gardy, J. (2020). Ethical challenges in the treatment of non-refugee migrants with tuberculosis in Canada. *Journal of Public Health*.

**Irrelevant Comparator (1)**

Gagnon, A. J., Dougherty, G., Wahoush, O., Saucier, J., Dennis, C., Stanger, E., . . . Stewart, D. E. (2013). International migration to Canada: The post-birth health of mothers and infants by immigration class. *Social Science & Medicine,* *76*, 197-207. doi:10.1016/j.socscimed.2012.11.001

**Irrelevant Setting (1)**

Siddiqi, A., Zuberi, D., & Nguyen, Q. C. (2009). The role of health insurance in explaining immigrant versus non-immigrant disparities in access to health care: Comparing the United States to Canada. *Social Science & Medicine,* *69*(10), 1452-1459. doi:10.1016/j.socscimed.2009.08.030

**Excluded Studies Identified Via Other Methods (5)**

Magalhaes, L., Carrasco, C., & Gastaldo, D. (2009). Undocumented Migrants in Canada: A Scope Literature Review on Health, Access to Services, and Working Conditions. *Journal of Immigrant and Minority Health,* *12*(1), 132-151. doi:10.1007/s10903-009-9280-5

Khanlou, N., Haque, N., Skinner, A., Mantini, A., & Landy, C. K. (2017). Scoping Review on Maternal Health among Immigrant and Refugee Women in Canada: Prenatal, Intrapartum, and Postnatal Care. *Journal of Pregnancy,2017*, 1-14. doi:10.1155/2017/8783294

Salami, B., Meharali, S., & Salami, A. (2015). The health of temporary foreign workers in Canada: A scoping review. *Canadian journal of public health*, *106*(8), e546-e554.

Gushulak, B. D., Pottie, K., Roberts, J. H., Torres, S., & DesMeules, M. (2011). Migration and health in Canada: health in the global village. *Cmaj*, *183*(12), E952-E958.

Bobadilla, A., Orchard, T., Magalhães, L., & Fitzsimmons, D. (2016). Ontario Healthcare Coverage Eligibility Among New Permanent Residents: A Scoping Review. *Journal of Immigrant & Refugee Studies,* *15*(4), 384-405. doi:10.1080/15562948.2016.1214993

**Appendix III: Screening and Extraction Forms**

The protocol for the study was not published in The International Prospective Register of Systematic Reviews (PROSPERO) as suggested by the PRISMA checklist as the project began as a student mini-review, which is an exclusion criteria in PROSPERO.

**1) Title and Abstract Screening Form**

| **Question** | **Options** | **Definitions / Additional Notes** |
| --- | --- | --- |
| Study Citation | Author(s): ______  Journal: ______  Year: ______ |  |
| Does the article:   1. Provide data on uninsured populations? 2. Have an outcome relevant to Medicare, health services utilization and/or cost of health care? 3. Provide Canadian results on provincially medically uninsured populations? 4. Provide primary quantitative data? | - Yes - No - Maybe | Examples of **uninsured** populations include but are not limited to refugees, asylum seekers, international students, temporary foreign workers and migrants who do not have a health card.  **Medicare** refers to Canada’s publicly funded health care system that allows Canadian residents to access medically necessary physician and hospital services without paying out of pocket  Health service outcome measurements deemed not medically necessary include dental care, vision care, chiropractic care, and prescription use.  At this stage, “**data**” refers to either primary or secondary data. |

**2) Full-Text Review Screening Form**

| **Question** | **Options** | **Definitions / Additional Notes** |
| --- | --- | --- |
| Does the article provide data on uninsured populations? | - Yes - No (Exclude, submit form without answering following questions) - Unsure (Exclude) | Examples of **uninsured** populations include but are not limited to refugees, asylum seekers, international students, temporary foreign workers and migrants who do not have a health card. To select yes, the article must specify clearly that they are studying uninsured groups/individuals. This also includes migrants in Canada who are uninsured for dental care/vision care at this stage of the form.  At this stage, “**data**” refers to either primary or secondary data. |
| **Only answer the following question if the answer to above question was Yes** | | |
| Does the article have an outcome or exposure measurement relating to:  a) Clinical outcome(s)  b) Medicare  c) Health services utilization  d) Cost of health care | - Yes, specify: ______ - No (Exclude, submit question without answering following questions) - Unsure (Exclude) | **Medicare** refers to Canada’s publicly funded health care system that allows Canadian residents to access medically necessary physician and hospital services without paying out of pocket  Health service outcome measurements deemed not medically necessary include dental care, vision care, chiropractic care, and prescription use. |
| **Only answer the following question if the answer to questions 1-2 was Yes** | | |
| Does the article provide Canadian results on provincially medically uninsured populations? | - Yes - No (Exclude, submit form without answering following question) - Unsure (Exclude) | To say Yes, there must be data on **provincially medically uninsured** populations in Canada |
| **Only answer the following question if the answer to questions 1-3 was Yes** | | |
| Does the article provide primary quantitative data? | - Yes, primary peer-reviewed paper (quantitative) - Yes, grey literature with primary data (government or research reports) - Yes, conference proceeding with sufficient detail - No, primary peer-reviewed paper (qualitative) (Exclude) - No, literature review (Exclude) - No, systematic review (Exclude) - No, grey literature without primary data (Exclude) - No, conference proceeding without sufficient detail (Exclude) - Unsure (Exclude) | If there is any quantitative data (for example mixed methods study), select **Primary peer-reviewed paper, quantitative**  **Systematic review:** Includes systematic review, scoping review, and meta-analyses  **Grey literature without primary data**: Includes newspaper or magazine articles |

**3) Data Extraction Form**

Note: Not all questions apply.

| **Question** | **Options** | **Definitions / Additional Notes** |
| --- | --- | --- |
| **General Questions** | | |
| Study Citation: |  |  |
| What is the study design? | Specify: _______ | **Observational study** - Assignment of subjects into a treatment group versus a control group is outside the control of the investigator   - **Cross-sectional -** Examines the relationship of a risk factor and outcome (disease) at a point in time on representative samples of the target population - **Cohort study -** Individuals with differing exposures to a suspected risk factor are observed through time for the occurrence of an outcome - **Case-control study** - Compares exposure to the risk factor in subjects who have an outcome (the 'cases') with subjects who do not have the outcome, but are otherwise similar (the 'controls') and drawn from the same sampling frame - **Case or case-series -** Descriptive study of a single individual (case report) or small group (case series) - **Longitudinal study -** Data is gathered for the same subjects over a period of time   **Experimental study -** Assignment of subjects to a treatment group or a control group before the start of an intervention   - **Control trial -** Experimental study in which people are allocated to intervention groups and evaluated for outcomes - **Quasi-experiment -** Experiment in which subjects are not randomly assigned to groups. Often this is the method of choice in field trials where the samples of the outcome are taken from the same individuals before and after the experiment/intervention   **Mixed methods -** Tackles a research question using different research methodologies. Please specify the research methodologies used |
| What is the year of publication? | Specify: ______ |  |
| What are the years of data collection? | Specify: ______ |  |
| Did the study provide definition of “medically uninsured” | Specify: ______ |  |
| How was the data collected? | Describe data collection process: ______ |  |
| **Study Participants** | | |
| Where was the study population-of-interest from?  (Select all that apply) | Province, specify: ______  City, specify: ______  Community, specify: ______ | **Provinces** refers to one of the ten provinces or three territories in Canada  **City** refers to a jurisdiction within a province  **Community** refers to a group of people who share a particular characteristic |
| Did the study include a control/comparison group? | - Yes - Concurrent comparison, specify: ______ - Pre-post comparison, specify: ______ - No | **Pre-post comparisons -** i.e. uncontrolled before-after studies |
| How were the participants selected? How were cases and controls selected? | Describe sampling strategy: ______ | **Specify database used** |
| What medically uninsured groups were studied?  (Select all that apply) | Specify cases and controls: ______ |  |
| Was private insurance studied? How so? | - Yes, specify: ______ - No - Not reported |  |
| What is the sample size (n) of the study? | Total (n): ______  Cases: ______  Controls: ______  Subgroups: ______ |  |
| Was the response/participation rate captured? | - Yes, specify: ______ - No - Not reported |  |
| Indicate the number of men and women in the study: | Specify number of men in medically uninsured group: _______  Specify number of women in medically uninsured group: _______  Specify number of men in comparison group: _______  Specify number of women in comparison group: _______ |  |
| Indicate the age of population  investigated: | Medically uninsured group: ______  Comparison group: ______ |  |
| Indicate the ethnicity of population investigated: | Medically uninsured group:   - Asian - Latino - Middle Eastern - Hispanic - African - Not reported - Other: ______   Comparison group:   - Asian - Latino - Middle Eastern - Hispanic - African - Not reported - Other: ______ |  |
| Indicate the country of origin of the population investigated: | Specify: ______ |  |
| Was any additional demographic information captured in the study results? If so, please specify. | Specify: ______ | Capture all of the details provided |
| Describe data analysis method: | Specify: ______ | Capture all of the details provided |
| **Data Extraction (DE)** | | |
| What category does the result fall under? | Clinical outcomes, specify: ______  Healthcare utilization, specify: ______  Cost of care, specify: ______ |  |
| **Cost of Care** | | |
| Were cost-estimates provided for the patient population/individual studied? | - Yes, cost to the individual patient - Specific cost estimates/numbers: ______ - Description: ______ - Yes, cost to the healthcare system - Specific cost estimates/numbers: ______ - Description: ______ - No |  |
| **Clinical Outcomes** | | |
| Was being medically uninsured studied as a risk factor for negative health outcomes? | Results on health: ______  Results on healthcare utilization: ______  Results on cost: ______ |  |
| If poor outcomes were reported, what demographic characteristics were studied as risk factors? | Age, specify: ______  Sex, specify: ______  Education, specify: ______  Marital status, specify: ______  Household income, specify: ______  Other, specify: ______ |  |
| What health outcomes were investigated in the medically uninsured population? | Cardiovascular-related, specify: ______  Accident, specify: ______  Maternal care, specify: ______  Chronic disease / illness, specify: ______ |  |
| What health outcomes were reported in the medically uninsured population? | Cardiovascular-related, specify: ______  Respiratory-related, specify: ______  Maternal / perinatal care, specify: ______  Emergency care, specify: ______  Sexual / reproductive health, specify: ______  Occupational health, specify: ______  Child / adolescent health, specify: ______  Injury / Accident, specify: ______  Other, specify: ______ | Specify estimates: Number of cases reported, percentages, prevalence |
| **Health Services Utilization** | | |
| What healthcare service was accessed by the medically uninsured population/individual? | Acute emergency care, specify: ______  Out-patient services, specify: ______  In-patient care/hospitalization, specify: ______ |  |
| Was the number/amount of times that the service was accessed reported? | Specify: ______ |  |
| Was the duration of healthcare utilization reported? | Specify: ______ |  |
| Was the severity of healthcare utilization reported? | Specify: ______ |  |
| **Final Questions** | | |
| What are the reported limitations of the study? | Limited by data source, specify: ______  Lack of knowledge on underdiagnosed/undertreated, specify: ______  Small sample size, specify: ______  Limited matching, specify: ______  Presence of bias, specify: ______  Ethical concerns, specify: ______ |  |

**Appendix IV: PRISMA 2020 Checklist**

| **Section and Topic** | **Item #** | **Checklist item** | **Location where item is reported** |
| --- | --- | --- | --- |
| **TITLE** | | |  |
| Title | 1 | Identify the report as a systematic review. | Title, Abstract, Methods |
| **ABSTRACT** | | |  |
| Abstract | 2 | See the PRISMA 2020 for Abstracts checklist. | Pg 1 |
| **INTRODUCTION** | | |  |
| Rationale | 3 | Describe the rationale for the review in the context of existing knowledge. | Pg 2-5 |
| Objectives | 4 | Provide an explicit statement of the objective(s) or question(s) the review addresses. | Pg 4-5 |
| **METHODS** | | |  |
| Eligibility criteria | 5 | Specify the inclusion and exclusion criteria for the review and how studies were grouped for the syntheses. | Pg 6 |
| Information sources | 6 | Specify all databases, registers, websites, organisations, reference lists and other sources searched or consulted to identify studies. Specify the date when each source was last searched or consulted. | Pg 5-6; Figure 1, Appendix |
| Search strategy | 7 | Present the full search strategies for all databases, registers and websites, including any filters and limits used. | Appendix |
| Selection process | 8 | Specify the methods used to decide whether a study met the inclusion criteria of the review, including how many reviewers screened each record and each report retrieved, whether they worked independently, and if applicable, details of automation tools used in the process. | Pg 6-7 |
| Data collection process | 9 | Specify the methods used to collect data from reports, including how many reviewers collected data from each report, whether they worked independently, any processes for obtaining or confirming data from study investigators, and if applicable, details of automation tools used in the process. | Pg 6-7 |
| Data items | 10a | List and define all outcomes for which data were sought. Specify whether all results that were compatible with each outcome domain in each study were sought (e.g. for all measures, time points, analyses), and if not, the methods used to decide which results to collect. | Pg 7, Appendix |
|  | 10b | List and define all other variables for which data were sought (e.g. participant and intervention characteristics, funding sources). Describe any assumptions made about any missing or unclear information. | Pg 7, Appendix |
| Study risk of bias assessment | 11 | Specify the methods used to assess risk of bias in the included studies, including details of the tool(s) used, how many reviewers assessed each study and whether they worked independently, and if applicable, details of automation tools used in the process. | Pg 6-7 |
| Effect measures | 12 | Specify for each outcome the effect measure(s) (e.g. risk ratio, mean difference) used in the synthesis or presentation of results. | Tables 3-4 |
| Synthesis methods | 13a | Describe the processes used to decide which studies were eligible for each synthesis (e.g. tabulating the study intervention characteristics and comparing against the planned groups for each synthesis (item #5)). | Pg 6-7 |
|  | 13b | Describe any methods required to prepare the data for presentation or synthesis, such as handling of missing summary statistics, or data conversions. | NA |
|  | 13c | Describe any methods used to tabulate or visually display results of individual studies and syntheses. | Pg 7 |
|  | 13d | Describe any methods used to synthesize results and provide a rationale for the choice(s). If meta-analysis was performed, describe the model(s), method(s) to identify the presence and extent of statistical heterogeneity, and software package(s) used. | Pg 7 |
|  | 13e | Describe any methods used to explore possible causes of heterogeneity among study results (e.g. subgroup analysis, meta-regression). | NA |
|  | 13f | Describe any sensitivity analyses conducted to assess robustness of the synthesized results. | NA |
| Reporting bias assessment | 14 | Describe any methods used to assess risk of bias due to missing results in a synthesis (arising from reporting biases). | Pg 7 |
| Certainty assessment | 15 | Describe any methods used to assess certainty (or confidence) in the body of evidence for an outcome. | Pg 7 |
| **RESULTS** | | |  |
| Study selection | 16a | Describe the results of the search and selection process, from the number of records identified in the search to the number of studies included in the review, ideally using a flow diagram. | Pg 7-8 |
|  | 16b | Cite studies that might appear to meet the inclusion criteria, but which were excluded, and explain why they were excluded. | Appendix |
| Study characteristics | 17 | Cite each included study and present its characteristics. | Table 1 |
| Risk of bias in studies | 18 | Present assessments of risk of bias for each included study. | Table 2 |
| Results of individual studies | 19 | For all outcomes, present, for each study: (a) summary statistics for each group (where appropriate) and (b) an effect estimate and its precision (e.g. confidence/credible interval), ideally using structured tables or plots. | Tables 3-4 |
| Results of syntheses | 20a | For each synthesis, briefly summarise the characteristics and risk of bias among contributing studies. | Tables 1-2, Pg 8-15 |
|  | 20b | Present results of all statistical syntheses conducted. If meta-analysis was done, present for each the summary estimate and its precision (e.g. confidence/credible interval) and measures of statistical heterogeneity. If comparing groups, describe the direction of the effect. | NA |
|  | 20c | Present results of all investigations of possible causes of heterogeneity among study results. | NA |
|  | 20d | Present results of all sensitivity analyses conducted to assess the robustness of the synthesized results. | NA |
| Reporting biases | 21 | Present assessments of risk of bias due to missing results (arising from reporting biases) for each synthesis assessed. | Table 2, Pg 8-15 |
| Certainty of evidence | 22 | Present assessments of certainty (or confidence) in the body of evidence for each outcome assessed. | Tables 2, Pg 8-15 |
| **DISCUSSION** | | |  |
| Discussion | 23a | Provide a general interpretation of the results in the context of other evidence. | Pg 16-18 |
|  | 23b | Discuss any limitations of the evidence included in the review. | Table 1 |
|  | 23c | Discuss any limitations of the review processes used. | Pg 18-19 |
|  | 23d | Discuss implications of the results for practice, policy, and future research. | Pg 19-20 |
| **OTHER INFORMATION** | | |  |
| Registration and protocol | 24a | Provide registration information for the review, including register name and registration number, or state that the review was not registered. | Appendix |
|  | 24b | Indicate where the review protocol can be accessed, or state that a protocol was not prepared. | Appendix |
|  | 24c | Describe and explain any amendments to information provided at registration or in the protocol. | NA |
| Support | 25 | Describe sources of financial or non-financial support for the review, and the role of the funders or sponsors in the review. | Title page |
| Competing interests | 26 | Declare any competing interests of review authors. | Title page |
| Availability of data, code and other materials | 27 | Report which of the following are publicly available and where they can be found: template data collection forms; data extracted from included studies; data used for all analyses; analytic code; any other materials used in the review. | Appendix, Methods |

**Appendix V: Completed data extraction forms**

| **Question** | **Response** |
| --- | --- |
| **Study Characteristics** | |
| **Title** | The negative self-perceived health of migrants with precarious status in Montreal, Canada: A cross-sectional study |
| **Journal** | PLOS ONE |
| **Authors and Year of Publication** | Cloos, P., Ndao, E.M., Aho, J., Benoît, M., Fillol, A., Munoz-Bertrand, Ouimet. M., Hanley, J., & Ridde, V., 2020. |
| **Study Objectives** | To examine the association between precarious migration status and self-perceived health in Montreal, Canada |
| **Study Design** | Cross sectional |
| **City and Province** | Montreal (Quebec) |
| **Data Collection Time Frame** | June 2016 to September 2017 |
| **Follow-Up Time Period** | N/A |
| **Data Collection Methods** | -Administered a questionnaire through 30-90 minute face-to-face interviews using a tablet to 806 individuals on the following: sociodemographic, socioeconomic and psychosocial characteristics, social determinants, health needs and access to health care, and health self-perception  -6 subsequent focus groups to discuss questions that were unclear or uncomfortable to answer |
| **Population Characteristics** | |
| **Total Sample Size** | n=806 |
| **Comparator / Control Group** | N/A |
| **Sample Selection Methods** | -Venue based community sampling methodology using snowball sampling and a local media (social, community, press) campaign (54.1% recruited in urban spaces)  -Recruitment through NGO Doctors in World (DoW)’s health clinic in Montreal (45.9% recruited in clinics) |
| **Uninsured Definition** | -Not having health insurance (Quebec's provincial health insurance RAMQ), IFHP or private insurance) |
| **Sex Distribution (% Male)** | 29.9% authorized migration status  44% unauthorized migration status |
| **Sex Distribution (% Female)** | 70.1% authorized migration status  56% unauthorized migration status |
| **Age Distribution** | Median age: 37  Range: 18-87 |
| **Ethnicity / Source Country** | Authorized migrant: 15.5% Sub-Saharan Africa; 35.2% Latin America; 5.5% Asia; 7.9% Caribbean; 1.2% United States; 8.9% Europe; 25.8% Middle East  Unauthorized migrant: 16.5% Sub-Saharan Africa, 36.4% Latin America, 4.3% Asia, 21.1% Caribbean, 0.9% United States, 5.4% Europe, 15.4% Middle East |
| **Other Baseline Demographics or Characteristics** | -Marital status  -Region of birth  -Living with children  -Duration of stay in Quebec  -Education  -Main occupation  -Work contract  -If family income meets needs  -Housing  -Food security  -Perceived discrimination  -Perceived fear  -Perceived stress |
| **Participant Eligibility Criteria** | -Born outside of Canada, reporting not having health insurance (whether RAMQ, IFHP, private insurance), being over 18 years of age, and residing in or intending to reside in the province of Quebec for more than 6 months or intending to obtain permanent residency |
| **Results: Cost** | |
| **Were cost-estimates provided for the patient population / individual studied?** | No |
| **Results** | N/A |
| **Results: Health Outcomes** | |
| **Was being medically insured studied as a risk factor for negative health outcomes?** | Yes |
| **If poor health outcomes were reported, what demographic characteristics were studied as risk factors?** | -Uninsured status  -Sociodemographic characteristics  -Socioeconomic characteristics  -Psychological characteristics |
| **What health outcomes were reported in the medically uninsured population?** | -Self perceived health (bad, fair, good, very good, excellent) was reported as well as other potential correlates for example, mental distress using the Kessler  -Psychological distress scale, previous diagnosis of a health problem (Y/N), health problem in past 12 months (Y/N), occupational injury (Y/N), number of years without health insurance, unmet health needs since without health insurance (Y/N) |
| **Results** | -527 (68.9%) uninsured migrants reported unmet health needs  -348 (44.6%) of all migrants perceived their health as negative  -192 (26.3%) reported mental distress  -652 (83.5%) of participants had a health problem within the past 12 months |
| **Utilization Outcomes** | |
| **What healthcare service was accessed by the medically uninsured population / individual?** | N/A |
| **Was the number / amount of times that the service was accessed reported?** | No |
| **Was the duration of healthcare utilization reported?** | No |
| **Results** | N/A |
| **Conclusion** | -The findings of this study suggest health perception among uninsured migrants in Montreal, Canada is poor compared to the Canadian-born and Canadian immigrant population as indicated in previous Canadian studies and Canadian Community Health Surveys (CCHS) data  -Authors conclude access to resources, healthcare and social services for all is imperative for migrant well-being and public health |
| **Limitations** | - Potentially unrepresentative sample (difficult to ascertain due to lack of a sampling frame)  -Recruitment in a clinic for uninsured (could introduce selection bias)  -Self reporting (could introduce social desirability bias)  -Cross sectional study design makes it impossible to establish causality  -Potential for mis reporting (information bias)  -Lack of statistical power  -Potential for missing confounding variables |
| **Strengths** | -Large sample size  -Recruitment process of uninsured individuals consisted of multiple avenues  -Pre-tested questionnaire in 6 languages with 5 participants for each language  -Utilized an established model to create questionnaire  -Pre-tested survey and conducted focus group to determine appropriateness of questionnaire |

| **Question** | **Response** |
| --- | --- |
| **Study Characteristics** | |
| **Title** | Unmet healthcare needs among migrants without medical insurance in Montreal, Canada |
| **Journal** | Global Public Health |
| **Authors and Year of Publication** | Ridde, V., Aho, J., Ndao, E.M., Benoit, M., Hanley, J., Lagrange, S., Fillol, A., Raynault, M.F., & Cloos, P., 2020. |
| **Study Objectives** | To examine the unmet healthcare needs and its associated factor among medicare uninsured migrants residing in Montreal, Canada |
| **Study Design** | Cross sectional survey |
| **City and Province** | Montreal (Quebec) |
| **Data Collection Time Frame** | June 2016 to September 2017 |
| **Follow-Up Time Period** | N/A |
| **Data Collection Methods** | -Eligibility was assessed using an informal screening questionnaire that asked about the trajectory of migration and healthcare coverage  -Assistants administered the questionnaire through face-to-face interviews |
| **Population Characteristics** | |
| **Total Sample Size** | n= 806  [2 accepted refugees, 57 asylum seekers, 30 temporary foreign workers and dependents, 68 students and dependents, 217 visitors, 104 other temporary residents, 141 no legal migrant status (application submitted), 161 no legal migrant status (no application submitted)]  Community= 436  NGO Clinic= 370 |
| **Comparator / Control Group** | N/A |
| **Sample Selection Methods** | -Venue-based sampling in the community where key informants from community organizations and academia came together and identified neighbourhoods and places that the study population was known to gather  -Participants in these meetings were encouraged to communicate with eligible participants  -Second recruitment at a NGO clinic in Montreal where patients were approached in waiting room  -The migrant clinic of DoW offers essential care for specific medical problems, psychosocial support services, information on rights and facilitates possible referral to other relevant care services but does not offer prenatal or other follow up services |
| **Uninsured Definition** | -Studied migrants not covered by the Province of Quebec public health insurance (RAMQ) or the Interim Federal Health Program (IFHP)  -Defined migrants as those born outside of Canada |
| **Sex Distribution (% Male)** | 294 (36.6%) |
| **Sex Distribution (% Female)** | 506 (63.0%) |
| **Age Distribution** | Mean= 40.3  Median= 37.0 |
| **Ethnicity / Source Country** | 15.2 % African  36.1% Latin America  5.2% Asia  13.9% Caribbean  8.5% European USA  21.1% Middle Eastern |
| **Other Baseline Demographics or Characteristics** | -Place of recruitment  -Education  -Marital status  -Region of origin  -Migratory status  -Occupation  -Duration of stay in Quebec  -Number of years without healthcare coverage |
| **Participant Eligibility Criteria** | -Uninsured (based on definition), over 18, resided or intended to reside in Quebec for more than 6 months and/or obtain permanent residence  -Individuals who were unaware that they were eligible for the IFHP or who had benefited from it in the past but had not been able to extend or to renew it were also included  -Exclusion criteria included: benefitting from private insurance that covered all types of primary care, being a Canadian citizen or a permanent resident; being under18 years old; being unable to communicate in one of the six languages of the study; and having already participated in the current study |
| **Results: Cost** | |
| **Were cost-estimates provided for the patient population / individual studied?** | No |
| **Results** | N/A |
| **Results: Health Outcomes** | |
| **Was being medically insured studied as a risk factor for negative health outcomes?** | Yes |
| **If poor health outcomes were reported, what demographic characteristics were studied as risk factors?** | -Education  -Migratory legal status  -Duration of stay  -Fluency in French  -Work Status  -Relationship/support  -Private Insurance  -Years without healthcare coverage  -Occupational Injury  -Diagnosed with health issue  -Sex |
| **What health outcomes were reported in the medically uninsured population?** | -Among participants who reported receiving medical diagnosis by a health care professional, the most frequently reported diagnosis included cardiovascular and circulatory disease, mental health issues, endocrine system, and musculoskeletal system |
| **Results** | -Compared to recent immigrants or the citizens with health care coverage, over 69.0% (two-third) of uncovered migrants participants reported that they had unmet health care needs  -This proportion was highest among migrants without health care coverage (69.0%) vs their insured counterparts (26% among insured immigrant and 16% among insured Canadian citizen)  -Over one-third (36.9%) of the migrant participants reported receiving a diagnosis by a healthcare professional; among these individuals the most frequently reported diagnosis included cardiovascular and circulatory disease (n = 99, 33.6%), mental health issues s (n = 40, 13.6%), endocrine system (n = 76, 25.8%), and musculoskeletal system (n = 33, 11.2%) |
| **Utilization Outcomes** | |
| **What healthcare service was accessed by the medically uninsured population / individual?** | -Private pharmacies  -Community organization health services  -Walk-in clinics  -Dental Clinics  -Hospitals |
| **Was the number / amount of times that the service was accessed reported?** | Not reported |
| **Was the duration of healthcare utilization reported?** | Not reported |
| **Results** | -Compared to recent immigrants (26%) or the citizens (16%) with health health care coverage, over 69.0% (two-third) of uncovered migrants participants reported that they had unmet health care needs. The unmet need was greater among temporary workers and their descendents (73%). The proportion with unmet health care needs were 73.3% (temporary foreign workers and dependents), 73.1% (students and dependents), 70.6% (other temporary residents), 63.5% (visitors), 62.5% (refugee claimants), 50% (accepted refugees), 67.4% (no legal migrant status with application submitted), and 76.4% (no legal migrant status with no application submitted)  -The association between unmet health care needs and migrant status was not statistically significant  -Reasons for unmet health care need included not having enough money to pay fees (81%), fear of being overcharged (73%), potential negative impact of health consultation on migration status (22%), and fear of rejection by hospital (7%)  -Almost a fifth (19%) reported not knowing where one can access health care  -Among those who used health care services, they used private pharmacies (60%), community organizations health services (43%), walk-in-clinics (21%), dental clinics (16%), and hospitals (14%). Services such as osteopathy, chiropractic, and physiotherapy was used by less than 3% |
| **Conclusion** | -There are many challenges in access to healthcare for migrants without healthcare coverage |
| **Limitations** | -Certain social groups (Chinese, Anglo-Caribbean migrants) underrepresented  -Participants could have been surveyed twice given that not identifying information was required  -Interviewer bias is possible due to self-reported data  -Only focused on migrants so didn't study how lack of health care coverage is associated with unmet health care needs |
| **Strengths** | Not reported |

| **Question** | **Response** |
| --- | --- |
| **Study Characteristics** | |
| **Title** | Outcomes of uninsured midwifery clients in Ontario, Canada: A retrospective cohort study |
| **Journal** | Midwifery |
| **Authors and Year of Publication** | Darling, E.K., Bennett, N., Burton, N., & Marquez, O., 2019. |
| **Study Objectives** | To analyze the characteristics, health service utilization, and clinical outcomes of Ontario residents not covered by OHIP that receive services from midwives |
| **Study Design** | Retrospective cohort |
| **City and Province** | Ontario |
| **Data Collection Time Frame** | April 1, 2012 - March 31, 2015 |
| **Follow-Up Time Period** | Pregnancy to 6-weeks postpartum |
| **Data Collection Methods** | -Analyzed BORN-Ontario data which provides information on all midwifery clients  -Data collection through BORN-Ontario is mandatory and is connected to midwives invoicing (ensures complete capture of all births and high completion of most variables)  -Data through BORN-Ontario is either uploaded directly from hospital electronic health records or entered into the online system by health care providers (enhances rigour of data collection strategy) |
| **Population Characteristics** | |
| **Total Sample Size** | n= 55, 634  Insured= 51,611  Uninsured= 4,023 |
| **Comparator / Control Group** | n= 51,611 (insured by OHIP) |
| **Sample Selection Methods** | -Using BORN-Ontario data, uninsured and insured participants were identified  -BORN-Ontario has built in data validation rules, quality checks and data verification processes  -Examined proportion of uninsured clients by practice group and by local health integration network (LHIN)  -Inclusion criteria: billed by a midwife as a course of care  -Exclusion criteria: clients with unknown insurance status, pregnancy loss prior to 20 weeks or pregnancy termination |
| **Uninsured Definition** | -Uninsured included those without publicly funded health insurance at provincial level  -This included landed immigrants in the 3-month waiting period for public health insurance, those with failed refugee claims who have not been deported, those who have overstayed their work visas, undocumented migrants, people with precarious living situations who have lost or stolen health cards and religious groups that have opted out of provincial coverage |
| **Sex Distribution (% Male)** | 0% |
| **Sex Distribution (% Female)** | 100% |
| **Age Distribution** | Mean age not provided  Sampled participants from <20, 20-24, 25-29, 30-34, 35-39 and 40+  Uninsured group consisted of a larger number of young participants than insured group |
| **Ethnicity / Source Country** | Not reported |
| **Other Baseline Demographics or Characteristics** | -Age  -Parity  -Pre-existing health conditions and obstetrical complications were measured in addition to utilization and health outcomes  -The local health integration network that the client is a part of |
| **Participant Eligibility Criteria** | -Billed by a midwife as a course of care (received at least 12 weeks of care from a midwife and/or had a midwife present at birth  -Excluded all clients with unknown in- surance status, a pregnancy loss prior to 20 weeks, or a pregnancy termination (any gestational age) |
| **Results: Cost** | |
| **Were cost-estimates provided for the patient population / individual studied?** | No |
| **Results** | N/A |
| **Results: Health Outcomes** | |
| **Was being medically insured studied as a risk factor for negative health outcomes?** | Yes |
| **If poor health outcomes were reported, what demographic characteristics were studied as risk factors?** | -Age  -Parity  -Pre-existing health conditions  -Obstetrical complications |
| **What health outcomes were reported in the medically uninsured population?** | -Gestational age at birth  -Mode of birth  -Type of labour  -Reasons for induction  -Fetal health surveillance  -Pharmacological pain management  -Intrapartum complications  -Preterm birth weight  -Rate of small for gestational age  -Exclusive breastfeeding at 6 months |
| **Results** | -Uninsured had higher rates of spontaneous labour (82.1% uninsured and 77.3% insured) and spontaneous vaginal birth (81.1% uninsured and 78.1% insured)  -Uninsured had lower rates of induction of labour(13.5% uninsured and 17% insured) , electronic fetal monitoring (12.6% uninsured and 14.6% insured), assisted vaginal birth (4.7% uninsured and 5.8% insured) and c-sections (13.9% uninsured and 15% insured)  -Uninsured more likely to use no pain medication during labour (46.7% uninsured and 37.1% insured). Insured more likely to use pain medication  -Postpartum hemorrhage (3.4% uninsured and 2.9% insured), preterm birth (5% uninsured and 4.4% insured) and small for gestational age (2.1% uninsured and 1.7% insured) more common in uninsured  -Exclusive breastfeeding at 6 weeks lower in uninsured (75.1% uninsured and 78.1% insured)  -Gestational age at birth was <29 weeks (0.5% uninsured and 0.3% insured), 29-33 weeks (0.7% uninsured and 0.7% insured), 34-36 weeks (3.9% uninsured and 3.4% insured), 37-38 weeks (18.9% uninsured and 17.8% insured), 39-41 weeks (74.7% uninsured and 76.2% insured) and >41 weeks (1.4% uninsured and 1.6% insured) |
| **Utilization Outcomes** | |
| **What healthcare service was accessed by the medically uninsured population / individual?** | -Antenatal services (first trimester visit, prenatal class, GBS screening, folic acid supplementation and women with at least one antenatal consultation with a physician)  -Intrapartum services  -Postpartum services (newborn metabolic screening, postpartum consultation with physician and transfer returned in postpartum) |
| **Was the number / amount of times that the service was accessed reported?** | No, but proportion of uninsured and insured accessing services was reported |
| **Was the duration of healthcare utilization reported?** | No |
| **Results** | -Uninsured attended fewer antenatal appointments than insured (mean 9.9 vs. 11.6 visits), 24.2% uninsured had at least one antenatal consultation with a physician while 30.3% in insured did  -Uninsured had more antenatal home visits than insured (mean 1.9 vists vs. 0.6 visits)  -Uninsured less likely to attend prenatal visit in first trimester (66.3% vs. 92.8%)  -Uninsured presented later to midwifery care (18.4 weeks gestation vs. 12.7 weeks)  -Uninsured less likely to attend prenatal class (33.2% vs. 65.2% for primiparous participants and 2.9% vs. 5.7% for multiparous participants  -33.9% uninsured planned home birth and 28.7% gave birth at home whereas 19.6% insured planned home birth and 16.6% gave birth at home  -61.2% uninsured planned hospital birth and 67.8% gave hospital birth, while 77.9% uninsured planned hospital birth and 81.9% did it. 3.3% uninsured planned hospital birth and 2% gave birth in birth centre, while 1.4% uninsured planned birth in birth centre and 0.7% did it  -0.6% uninsured planned clinic while 0.4% uninsured planned birth in clinic and none in either groups did  -0.6% uninsured planned birth in other locations and 1% gave hospital birth, while 0.5% uninsured planned hospital birth and 0.5% did it  -Uninsured had shorter hospital stays  -Uninsured received more postpartum home visits than insured clients (mean 3.7 visits vs. 3.2)  -EMS transfer while in labour was 2.61 for uninsured and 1.7% for insured  -30.6% uninsured had at least one intrapartum consultation with physician while 35.5% insured did  -Intrapartum transfer of care was 21.3% in uninsured and 23.8% in insured  -Transfer of care returned in labor was 6.1% in uninsured and 23.8% in insured  -Primary accoucher was registered midwife (64.7% uninsured and 60.2% insured), midwifery student (4.6% uninsured and 5.1% insured), obstetrician (28% uninsured and 32.6% insured), and family physician (0.8% uninsured and 0.6% insured)  -Level of hospital care for hospital births was 1 for 12.4% uninsured and 12.2% insured, 2 for 77.3% uninsured and 74.5% insured, and 3 for 10.6% uninsured and 13.4% insured  -NICU admissions were 8.7% in uninsured and 9.2% in insured  -Uninsured less likely to have at least one postpartum consultation with a physician (5.5% vs. 6.8%) |
| **Conclusion** | -Uninsured group had lower mean BMI (22.6) than insured clients (24.2)  -Uninsured group used less services than insured  -Uninsured had good clinical outcomes using midwifery services |
| **Limitations** | -BORN-Ontario registry may be subject to misclassifications, data entry errors and missing data (very low rates of missing data for the demographic variables and outcomes variables that were reported and audits show consistency with registry and clinical records)  -BORN-Ontario registry does not contain information about the reason why individuals are uninsured (could not distinguish between subgroups in the uninsured category)  -Did not do chi-square analysis to see whether the proportions differ by insurance status |
| **Strengths** | -Provides description of the demographics, clinical outcomes and service use of all women (insured and uninsured) using midwifery services in Ontario over three years  -Using an administrative dataset that focuses on midwives |

| **Question** | **Response** |
| --- | --- |
| **Study Characteristics** | |
| **Title** | Use of the emergency department by refugees under the Interim Federal Health Program: A health records review |
| **Journal** | PLOS ONE |
| **Authors and Year of Publication** | Bakewell, F., Addleman, S., Dickinson, G., & Thiruganasambandamoorthy, V. (2018) |
| **Study Objectives** | To examine the effect of the cuts on emergency department (ED) use among patients claiming IFHP benefits |
| **Study Design** | Retrospective chart review |
| **City and Province** | Ottawa, Ontario |
| **Data Collection Time Frame** | January 1, 2011 to December 31 2013 |
| **Follow-Up Time Period** | 18 months before and after the cuts |
| **Data Collection Methods** | Review of electronic medical record of the ED chart |
| **Population Characteristics** | |
| **Total Sample Size** | n=612 |
| **Comparator / Control Group** | Before n= 357  After n= 255 |
| **Sample Selection Methods** | All ED visits where a claim was made at triage for coverage under the IFHP, from January 1, 2011 to December 31, 2013 |
| **Uninsured Definition** | Refugee claimants who made claims under the IFHP at ED visit triage |
| **Sex Distribution (% Male)** | NR |
| **Sex Distribution (% Female)** | Before n= 202 (56%)  After n= 134 (52.5%) |
| **Age Distribution** | Median age before= 41  Median age after= 38 |
| **Ethnicity / Source Country** | NR |
| **Other Baseline Demographics or Characteristics** | -Canadian Triage and Acuity Score (CTAS)  -Chief presenting complaint  -Discharge diagnosis  -If the patient had a family doctor |
| **Participant Eligibility Criteria** | ED patients who had claimed IFHP coverage at triage |
| **Results: Cost** | |
| **Were cost-estimates provided for the patient population / individual studied?** | Average cost of rejected claim |
| **Results** | IFHP claims:  - There were 357 claims prior to cuts and 255 afterwards (28.6% reduction)  -Accounting for repeat visits, there were 201 claims prior to cuts and 148 after (26.4% reduction)  Physician costs:  -Rejected physician billing claims were significantly more after the IFHP cuts: (13.7% vs. 3.9%; OR 4.28 95% CI: 2.18-8.40; p<0.05)  -Fewer of the rejected claims for physician billings were paid by patients after the cuts  ( 20.0% vs. 50%; OR 4, 95% CI: 1.05–15.15; p = 0.076)  -Referment to collections agency decreased ( 20% vs. 50%)  -Physician billings before-and-after cuts were $131.14 and $115.76 CAD, respectively  Hospital costs:  -Hospital costs claims rejected was higher after the IFHP costs (7.5% vs. 1.1%; OR 4.28 95% CI: 2.39-21.15; p<0.05)  -Hospital cost paid by uninsured patients was less after the IFHP cuts compared to pre cuts (25% vs. 15.8%;  OR 1.78, 95% CI: 0.14–23.42)  -Referment to collections agency increased (84.2% vs. 75%)  -Hospital billings before-and-after cuts were $990.04 and $726.88 CAD, respectively  -Total costs of rejected claims was higher after cuts ($17,862.5 vs. $5,796.1 CAD) |
| **Results: Health Outcomes** | |
| **Was being medically insured studied as a risk factor for negative health outcomes?** | No |
| **If poor health outcomes were reported, what demographic characteristics were studied as risk factors?** | -Discharge diagnosis  -Hospital charges and payment status were also assessed for any claims that had been rejected by ED physician billing  -The financial status of IFHP claims for ED physician billings were reviewed  -Visit frequency |
| **What health outcomes were reported in the medically uninsured population?** | -Diagnosis  -Severity using the CTAS |
| **Results** | -The presentations and diagnoses of patients were similar before and after the IFHP cuts  -Illness severity as assessed by the Canadian Triage and Acuity Scale (CTAS) and by hospital admission rates were similar in the two groups |
| **Utilization Outcomes** | |
| **What healthcare service was accessed by the medically uninsured population / individual?** | -Emergency department  -Family physician |
| **Was the number / amount of times that the service was accessed reported?** | Yes |
| **Was the duration of healthcare utilization reported?** | No |
| **Results** | -IFHP coverage was claimed for 612 patient visits to the TOH ED during the study period  -There were 357 claims prior to the IFHP cuts and 255 after (a 28.6% reduction)  -Accounting for repeat visits, there were 201 individual patients claiming IFHP coverage before and 148 after (a 26.4%  reduction)  -Compared to pre-cuts, there was a significant reduction in access to family physician post-cuts (20.4% vs. 30%; OR =1.67,95% CI: 1.14–2.44; p = 0.009)  -The number of those without a designated family physician was significantly lower pre-cuts when compared to post-cuts:  41.8%  67.2%; OR= 2.8; 95% CI 1.45–5.62; p <0.003  -Even with reduced access to family physicians, higher proportion of patients though not significantly different, were advised to follow up with their family physician during their ER visit; 26.3% vs. 22.1%; (OR= 1.25, 95% CI: 0.86–1.82)  -Prescriptions were given to patients at a similar rate before and after the cuts (39.7% and 46.3%, p = 0.136)  -After the cuts, only 20.4% of patients had access to a family physician (FP) documented on their ED chart, compared to 30.0% before (OR 1.67, 95% CI: 1.14–2.44; p = 0.009) |
| **Conclusion** | -A higher proportion of both rejected and subsequently unpaid claims after the IFHP cuts represents a potential barrier to emergency medical care, as well as a new financial burden to be shouldered by patients and hospitals  -A reduction in IFHP claims in the ED and a reduction in the number of patients with access to a family physician also suggests inadequate primary care for this population  -Yet this was not reflected in the follow-up advice offered by ED physicians to patients |
| **Limitations** | -The study considers only those patients who made claims under the IFHP at triage, rather than all refugees seeking care  -It is therefore unknown if refugee claimants who had their IFHP coverage revoked still visited the ED as ‘self-pay’ patients, or if they avoided emergency care completely due to an inability to pau  -Some of the patients who had their claims rejected may not have been ineligible due to the changes in coverage, but may have had their refugee application denied  -Another limitation to the study is that older rejected claims (including those rejected before the cuts) have had more time to be paid by patients, and so differences in payment status may simply reflect the passage of time rather than ability to pay |
| **Strengths** | -Adjusted analysis  -The authors were able to identify that although the cut achieved its goal of saving some cost to the government, the saving was just a redistribution of cost from the government to refugees |

| **Question** | **Response** |
| --- | --- |
| **Study Characteristics** | |
| **Title** | Emergency Room Visits by Uninsured Child and Adult Residents in Ontario, Canada: What Diagnoses, Severity and Visit Disposition Reveal About the Impact of Being Uninsured |
| **Journal** | Journal of Immigrant and Minority Health |
| **Authors and Year of Publication** | Hynie, M., Ardern, C., & Robertson, A. (2016) |
| **Study Objectives** | To discuss the number of acute care visits to emergency rooms made to Ontario hospitals by residents without insurance and to compare the diagnoses, severity and outcomes associated with acute care visits by Ontario residents with and without insurance between 2002/3 and 2010/11 |
| **Study Design** | Cross sectional study on National Ambulatory Care Reporting System (NACRS) |
| **City and Province** | Ontario |
| **Data Collection Time Frame** | 2002 to 2003 and 2010 to 2011 |
| **Follow-Up Time Period** | N/A |
| **Data Collection Methods** | Data for 9 consecutive years (2002/2003–2010/2011) of the National Ambulatory Care Reporting System (NACRS) were obtained and used in the present analysis |
| **Population Characteristics** | |
| **Total Sample Size** | n= 44,489,750 (unique emergency department visits) |
| **Comparator / Control Group** | n= 140,730 (unique emergency department visits in the uninsured) |
| **Sample Selection Methods** | All emergency visits in the National Ambulatory Care Reporting System (NACRS) data |
| **Uninsured Definition** | Uninsured participants were identified as those individuals who were Canadian residents, who were self-paying, aged 80 years or less, and who reported a fixed address. There were a variety of ways in which individuals in Ontario became uninsured:  -New permanent residents,  -Those who have been living outside of the province who has to wait three months before they have access to health insurance  -Violations of temporary work permits  -Immigration sponsorship breakdown  -Overstaying visitor or work visas or  -Being a visitor  -Being a foreign student or  -Belonging to certain categories of temporary worker  -There are also those who are entitled to health insurance but lack documentation. For example, people who are homeless and/or lack secure housing can face challenges in safely storing their documents, and without these documents are often treated as if they were uninsured |
| **Sex Distribution (% Male)** | N/A |
| **Sex Distribution (% Female)** | Insured n= 50.5%  Uninsured n= 41.8% |
| **Age Distribution** | Insured mean=38.2  Uninsured mean= 31.4 |
| **Ethnicity / Source Country** | NR |
| **Other Baseline Demographics or Characteristics** | 4.8 % of all visits to emergency rooms were by children aged 16 or younger |
| **Participant Eligibility Criteria** | ED visit in Ontario:  -Only those associated with a Canadian postal code were included  -Those with homelessness status were excluded |
| **Results: Cost** | |
| **Were cost-estimates provided for the patient population / individual studied?** | No |
| **Results** | NR |
| **Results: Health Outcomes** | |
| **Was being medically insured studied as a risk factor for negative health outcomes?** | Yes |
| **If poor health outcomes were reported, what demographic characteristics were studied as risk factors?** | -The reporting facility  -Responsibility for payment  -Sociodemographic information about the patient (e.g., postal code of residence; birth date; gender)  -Information about the visit (e.g., whether arrived by ambulance; date and time when patient was registered; visit disposition; main problem; main intervention; triage level; date and time when a decision was made about the visit disposition) |
| **What health outcomes were reported in the medically uninsured population?** | -Frequencies for main diagnoses  -Severity (triage) of diagnosis  -mental health  -Injury, lab, Respiratory, Mental/behavioural, Social/environmental, Musculoskeletal/connective tissue, Digestive, Diseases of the eye, Genitourinary, Diseases of skin,  Obstetric, Hypertension, Asthma, Chronic obstructive pulmonary disease, Coronary heart failure, Diabetes or Angina |
| **Results** | -Notably, mental health/behavioural conditions were three times higher (uninsured: 10.5 %; insured: 3.5 %), and obstetric complications were twice as high (uninsured: 5.6 %; insured: 2.7 %) in the uninsured  -Most frequent main diagnoses by insurance type: injury (24.49% in insured vs. 28.37% in uninsured), other clinical/lab (18.40% in insured vs. 17.02 in uninsured), respiratory (11.21% in insured vs. 8.14%), social/environmental (7.49% and 7.18%) musculoskeletal/connective tissue (5.96% vs. 3.63%), digestive (5.88% vs. 5.38%), diseases of the eye (4.67% vs. 2.93%), genitourinary (4.50 vs. 4.50), diseases of the skin (3.60 vs 3.07), mental/behavioural (3.48% vs. 10.47%), obstetric (2.69% vs. 5.56%), and other (10.32% vs. 9.32%) |
| **Utilization Outcomes** | |
| **What healthcare service was accessed by the medically uninsured population / individual?** | Yes  -Visit disposition  -More ACSC visits were made by uninsured children and youth |
| **Was the number / amount of times that the service was accessed reported?** | No |
| **Was the duration of healthcare utilization reported?** | No |
| **Results** | -The percentage of visits of uninsured increased from 0.23 % in 2002/3 to 0.44 % in 2010/11  -Within Ontario, the proportion of visits by the uninsured to emergency departments ranged almost tenfold, from a low of 0.07 % in Erie St. Clair, to a high of 0.66 % in Toronto  -Visit disposition also differed by insurance status in that those without insurance were less likely to be admitted (insured: 10.2 %; uninsured: 9.4 %), more likely to leave without treatment (insured: 3.1 %; uninsured: 5.4 %), and more likely to have died on arrival or in the emergency department (insured: 2.8 %; uninsured: 3.7 %)  -Overall, ACSC related emergency visits were more common among the insured (4.55 %) than uninsured (3.18 %)  -However, a larger proportion of ACSC visits were accounted for by children (≤16 years), and youth (17–24 years) in the uninsured than insured group  - Those who were with insurance or not were equally likely to be triaged into one of the severe categories if they arrived with ACSC conditions  - At hospital presentation, 15.6 % of uninsured and 11.2 % of insured individuals were triaged into one of the more severe categories (Resuscitation or Emergent)  - For no ACSC, 10.5% and 25.8% insured and uninsured, respectively, were triaged to resuscitation, emergent. For ACSC, it was 49.8% and 54.1%  For no ACSC, 89.5% and 74.2% insured and uninsured, respectively, were triaged to urgent, less urgent, or non-urgent For ACSC, it was 50.2% and 45.9%  - Compared to insured individuals [odds ratio (OR) 1.00, referent], those who were uninsured were 43 % more likely (OR 1.43, 95 % CI 1.39–1.46) to be classified as severe, even after adjustment for age and gender |
| **Conclusion** | -Insurance status is associated with more serious health status on arrival to emergency departments and more negative visit outcomes |
| **Limitations** | -NACRS data represent visits, and not individuals  -It is possible that the numbers of uninsured are inflated by repeat visits by individuals who have no other access to health care  -It is also possible that, despite only including those individuals who were not identified as being homeless, some of the uninsured are in fact insurable but undocumented people experiencing homelessness not having legal status does not preclude experiencing homelessness, and thus some of those excluded due to homelessness may have been uninsured |
| **Strengths** | -large sample size |

| **Question** | **Response** |
| --- | --- |
| **Study Characteristics** | |
| **Title** | The cost and impact of the interim federal health program cuts on child refugees in Canada |
| **Journal** | PLOS ONE |
| **Authors and Year of Publication** | Evans, A., Caudarella, A., Ratnapalan, S., & Chan, K. (2014) |
| **Study Objectives** | To determine the financial and healthcare impact of IFHP funding cut on the refugee claimants |
| **Study Design** | Retrospective cohort study |
| **City and Province** | Toronto, Ontario |
| **Data Collection Time Frame** | January 1, 2012 to December 31, 2012 |
| **Follow-Up Time Period** | Visits included were six months prior and after IFHP cuts |
| **Data Collection Methods** | -Information on ER and admission data was collected from the Wellsoft Emergency Department Information System  -International Classification of Disease (10th edition) was obtained from patient hospital admission charts by two principal authors  -Billing information was obtained from the Accounts Receivables database of SickKids  -Billing information included the amount billed to the non-profit insurance company Medavie Blue Cross, who provides health insurance coverage for the IFHP, and the amount paid from Medavie Blue Cross to SickKids |
| **Population Characteristics** | |
| **Total Sample Size** | n= 315  Before cuts= 173  After cuts= 142 |
| **Comparator / Control Group** | 173 refugee claimants before the cuts |
| **Sample Selection Methods** | Included all ER visits at SickKids for children under 18 years of age |
| **Uninsured Definition** | N/A |
| **Sex Distribution (% Male)** | Before cuts= 47%  After cuts= 58% |
| **Sex Distribution (% Female)** | Before cuts= 53%  After cuts= 42% |
| **Age Distribution** | Mean age of before and after cuts groups= 6.8 |
| **Ethnicity / Source Country** | NR |
| **Other Baseline Demographics or Characteristics** | -Gender  -length of stay  -Canadian Triage Acuity Score |
| **Participant Eligibility Criteria** | -The study included data from all ER children visits who are less than 18 years of age with payment status classified as IFHP from Jan 1, 2012 to Dec 31, 2012  -Patients were classified as registered under the IFHP if they presented with IFHP papers, were refused claimants, refugee claimants ‘in process’ or pre-hearing  -Excluded participants who were uninsured, undocumented, arriving without IFHP papers, permanent immigrant, temporary worker or otherwise not identified as IFHP refugee claimants in the ER database |
| **Results: Cost** | |
| **Were cost-estimates provided for the patient population / individual studied?** | Yes |
| **Results** | -Total ER bill prior and after IFHP cuts was $20,010 and $13,549 respectively (p-value=0.74) with median being 93.70[93.70-93.70] before cuts and 93.70[93.70-93.70] after cuts  -Total admission bill prior and after IFHP cuts was $14912 and $73144 respectively with median bill being 1337.40[668.60-2006.10] before cuts and 1671.75[668.70-4666.68] after cuts  -Before IFHP cuts, 46% of total emergency room bills were paid by IFHP (compared to 7% after cuts)  -The total number of bills paid, and the total amount of money unpaid by Medavie Blue Cross insurance was significantly lower after the IFHP policy changes (p<0.01) - 93% ($12601) of the ER bills submitted by SickKids to Medavie Blue Cross insurance post-IFHP changes were unpaid, whereas 54% of the bills pre-IFHP changes were unpaid (10706)  -Number of bills unpaid post-cuts was 91% and prior the cuts was 57%. For admissions, 57% ($31483) of the ER bills submitted by SickKids to Medavie Blue Cross insurance post-IFHP changes were unpaid, whereas 60% of the bills pre-IFHP changes were unpaid (8894)  -Number of bills unpaid post-cuts was 82% and prior the cuts was 64% |
| **Results: Health Outcomes** | |
| **Was being medically insured studied as a risk factor for negative health outcomes?** | No |
| **If poor health outcomes were reported, what demographic characteristics were studied as risk factors?** | N/A |
| **What health outcomes were reported in the medically uninsured population?** | -The top three most responsible diagnoses by ICD-10 code for admission by refugee children during the study time period was sickle cell anemia with crisis (4), epilepsy (not intractable) (3), and appendicitis (2)  -There were no respiratory or viral illnesses in the child refugee population admitted  -The most common reason for admission for the general population in the same time period was pneumonia, supracondylar fracture of the humerus, and sickle cell anemia with crisis |
| **Results** | -No significant difference between age, gender, length of stay, or CTAS score before and after IFHP funding cuts or between refugee children and general population admitted to SickKids - 6.4% refugee claimants were admitted before cuts and 12% after the cuts (p=0.08) |
| **Utilization Outcomes** | |
| **What healthcare service was accessed by the medically uninsured population / individual?** | -Hospital  -Emergency room |
| **Was the number / amount of times that the service was accessed reported?** | N/A |
| **Was the duration of healthcare utilization reported?** | Yes, length of hospital stay if admitted was reported on |
| **Results** | -173 documented hospital visits by refugee children prior to the IFHP cuts and 142 visits in the six months after the cut  -Proportion of refugees presenting to ER after cuts significantly decreased (p<0.01; total admissions before cuts was 25755 prior to the cuts and 31189 after the cuts)  - The high acuity visits (CTAS 1 or 2) represented 20% and 19% of visits pre- and post-IFHP changes, respectively  -Admission length of stay in days was 2.8[1.6-3.0] for refugee claimants before cuts and 2.2[0.8-2.8] after cuts (p=0.32) |
| **Conclusion** | -IFHP cuts negatively impacted refugee children health by decreasing access to affordable health care services  -Policy changes were expected to save Canadian taxpayers $20 million annually  -Changes in funding IFHP provides savings to the federal level but transfers the cost to the institution treating the patient |
| **Limitations** | -Narrow range of data  -Small number of participants  -Only investigated patient experiences from a single institution  -Did not study a cohort of individuals who went from coverage to no-coverage (there were only 6 who showed a change in billing category from IFHP to uninsured) |
| **Strengths** | -Preliminary investigation into the IFHP cuts; showed that savings at the federal level transfer costs to hospitals |

| **Question** | **Response** |
| --- | --- |
| **Study Characteristics** | |
| **Title** | Understanding the demographic characteristics and health of medically uninsured patients |
| **Journal** | Canadian Family Physician |
| **Authors and Year of Publication** | Bunn, S., Fleming, P., Rzeznikiewiz D., & Leung, F. (2013) |
| **Study Objectives** | To determine demographic and diagnostic information about the medically uninsured patient population and compare it with that of the medically insured patient population at a primary care centre. |
| **Study Design** | Retrospective cohort |
| **City and Province** | Toronto, Ontario |
| **Data Collection Time Frame** | 2005 to 2009 |
| **Follow-Up Time Period** | N/A |
| **Data Collection Methods** | -Billing records from St. Michael's Hospital Department of Family and Community Medicine were analyzed to identify who was billed through the Compassionate Care Program from 2005 to 2009  -Medical chart audit to determine age, sex, postal code, if patient had a specific diagnosis, substance addiction, or mental health disorder, if patient accessed primary care and the reason for the patient’s uninsured status (if available) |
| **Population Characteristics** | |
| **Total Sample Size** | n= 95  Insured= 50  Uninsured= 45 (12 landed immigrant in 3-month waiting period, 10 no permanent residence in Canada, 7 lost or expired health card, 5 foreign visitor, 11 no reason provided) |
| **Comparator / Control Group** | Control group (insured) n= 50 |
| **Sample Selection Methods** | -Medical chart audit from 2005 to 2009 at St. Michael's Hospital Department of Family and Community medicine found 52 patients who were billed through the Compassionate Care Program and 45 were included  -52 insured patients were randomly selected and 50 were included. Excluded those whose charts could not be retrieved |
| **Uninsured Definition** | -Uninsured patients were those who were billed through the Compassionate Care Program  -This program was established by the Department of Family and Community Medicine at St. Michael's Hospital  -It waives hospital registration fees (about $200) and physician professional fees (about $40) for medically uninsured patients who cannot afford to pay out of pocket |
| **Sex Distribution (% Male)** | Insured n= 22 (44%)  Uninsured n= 18 (40%) |
| **Sex Distribution (% Female)** | Insured n= 28 (56%)  Uninsured n= 27 (60%) |
| **Age Distribution** | Insured mean= 40.3  Uninsured mean= 38.9 |
| **Ethnicity / Source Country** | N/A |
| **Other Baseline Demographics or Characteristics** | -Age  -Sex  -Mean income per household  -Diagnosis  -Category of primary care used  -Reason for being uninsured |
| **Participant Eligibility Criteria** | -Uninsured: any person using the Compassionate Care Program at St. Michael's Hospital from 2005 to 2009  -Insured: any person seeking care at St. Michael's Hospital from 2005 to 2009 with coverage from OHIP |
| **Results: Cost** | |
| **Were cost-estimates provided for the patient population / individual studied?** | No |
| **Results** | N/A |
| **Results: Health Outcomes** | |
| **Was being medically insured studied as a risk factor for negative health outcomes?** | Yes |
| **If poor health outcomes were reported, what demographic characteristics were studied as risk factors?** | -Age  -Sex  -Income |
| **What health outcomes were reported in the medically uninsured population?** | -If the patient had a specific diagnosis (hypertension, type 2 diabetes, HIV, tuberculosis, substance addiction or mental health disorder)  -If the patient accessed a specific type of care and the reason for uninsured status |
| **Results** | -Hypertension (14% insured 16% uninsured; p=0.831)  -Type 2 diabetes (6% insured 11% uninsured; p=0.470)  -HIV (4% insured 24% uninsured; p=0.004)  -Tuberculosis (6% insured 13% uninsured; p=0.300)  -Substance addiction (6% insured 4% uninsured; p>0.99)  -Mental health disorders (14% insured 16% uninsured; p=0.831) |
| **Utilization Outcomes** | |
| **What healthcare service was accessed by the medically uninsured population / individual?** | -Primary care (ie. prenatal or pediatric care) |
| **Was the number / amount of times that the service was accessed reported?** | No |
| **Was the duration of healthcare utilization reported?** | No |
| **Results** | -No significant difference between participants with OHIP vs. participants utilizing the Compassionate Care Program in the proportion of patients seeking prenatal or routine pediatric care  -6% and 16% of insured and uninsured used prenatal care, respectively (p=0.184)  -18% and 11% of insured and uninsured used routine pediatric care, respectively (p=0.344) |
| **Conclusion** | -Concluded that insured and uninsured patients do not differ significantly with prevalence of hypertension, type 2 diabetes, tuberculosis, substance addiction or mental health disorders, or the proportion of patients who access prenatal or routine pediatric care  -Most common reason for uninsurance was due to the three month waiting period  -Majority of uninsured who were HIV positive were uninsured because they did not have permanent resident status in Canada |
| **Limitations** | -Limited by sample size of the uninsured participants as only 52 utilized the Compassionate Care Program from 2005 to 2009  -Many patients accessing St. Michael's Hospital are vulnerable, inner city patients. Results may not be generalizable to a greater population  -Members of uninsured group were uninsured for a number of reasons; heterogeneous group  -Unknown if all participants were screened for all of the diagnoses investigated  -No control for confounding effects  -Internal validity of this study was limited by the fact that medical charts of 7 uninsured patients and 2 insured patients could not be located  -Not sure whether the two groups are comparable in all fronts except for insurance status, just compared income, age, and sex |
| **Strengths** | -Looked at chart data from the compassionate care program and insured and non-insured came from the same hospital |

| **Question** | **Response** |
| --- | --- |
| **Study Characteristics** | |
| **Title** | Predictors of emergency cesarean delivery among international migrant women in Canada. |
| **Journal** | International Journal of Gynecology and Obstetrics |
| **Authors and Year of Publication** | Gagnon, A.J., Merry, L., & Haase, K., 2013. |
| **Study Objectives** | To determine predictors of emergency caesarean delivery in order to develop a better understanding of disparities in emergency caesarean delivery rates between Canadian-born and migrant women |
| **Study Design** | Prospective cohort |
| **City and Province** | Toronto (Ontario), Montreal (Quebec), and Vancouver (British Columbia) |
| **Data Collection Time Frame** | February 1, 2006 - May 31, 2009 |
| **Follow-Up Time Period** | 1 and 4 weeks postpartum |
| **Data Collection Methods** | -Clinical data was obtained from medical records  -Additional information including migration status were obtained through interviewer-assisted questionnaires administered in postpartum hospital units at 1 and 4 weeks postpartum. |
| **Population Characteristics** | |
| **Total Sample Size** | N= 1025 migrant women  Refugees= 149  Asylum seekers= 371  Immigrants= 505 |
| **Comparator / Control Group** | Immigrants=505 |
| **Sample Selection Methods** | -Women were recruited from 12 Canadian hospitals in 3 cities through the Childbearing Health and Related Services Needs of Newcomers (CHARSNN) study  -Consecutive sampling was used for refugees and asylum seekers, and immigrant and Canadian-born women were alternately recruited based on matching date and time of birth (as closely as possible) to the refugees and asylum seekers included in the study |
| **Uninsured Definition** | Not defined |
| **Sex Distribution (% Male)** | 0% |
| **Sex Distribution (% Female)** | 100% |
| **Age Distribution** | Not reported |
| **Ethnicity / Source Country** | Not reported |
| **Other Baseline Demographics or Characteristics** | Not reported |
| **Participant Eligibility Criteria** | -Able to speak one of the study languages (Arabic, Dari or Persian, English, French, Mandarin or Cantonese, Punjabi, Russian, Serbo-Croatian, Somali, Spanish, Tamil, or Urdu)  -Being a refugee or asylum seeker within the past 5 years or immigrant  -Discharged from hospital within 4 days postpartum if delivered vaginally or 7 days postpartum if delivered via caesarean section  -Those with no known immigration status |
| **Results: Cost** | |
| **Were cost-estimates provided for the patient population / individual studied?** | No |
| **Results** | Not applicable |
| **Results: Health Outcomes** | |
| **Was being medically insured studied as a risk factor for negative health outcomes?** | Yes |
| **If poor health outcomes were reported, what demographic characteristics were studied as risk factors?** | -Parity (first delivery)  -Birth weight  -No health insurance  -Day of the week of delivery (Friday)  -Annual income < $30 000  -Induced labour  -Refugee  -Asylum seeker |
| **What health outcomes were reported in the medically uninsured population?** | -Emergency caesarean delivery  -Planned caesarean or vaginal delivery |
| **Results** | -Among migrant women, no health insurance coverage is associated with higher risk for emergency caesarean delivery (OR, 2.8; 95% CI, 1.2–6.3)  -Compared with immigrants, being an asylum seeker (0R, 0.3; 95% CI 0.2-0.6) or refugee (OR, 0.5; 95% CI, 0.2-1.0) was protective |
| **Utilization Outcomes** | |
| **What healthcare service was accessed by the medically uninsured population / individual?** | -Obstetric (labour, delivery and postpartum)  -Hospital care |
| **Was the number / amount of times that the service was accessed reported?** | No |
| **Was the duration of healthcare utilization reported?** | No |
| **Results** | Significant differences in NICU admission were found among the groups (refugee, 26.7%; asylum seeker, 15.6%; immigrant, 7.0%; p=0.073) |
| **Conclusion** | Absence of health insurance was predictive of higher emergency caesarean delivery rates in migrant women |
| **Limitations** | -Heterogeneity of comparison group  -Uninsured population is not defined – unsure whether it was refugees, asylum-seekers, or the immigrants  -No analysis of maternity unit characteristics  -Full regression results are not presented  -In original study, Canadian-born women were included but they did not act as comparator in this study |
| **Strengths** | -Followed-up women postpartum  -Looked at three different groups of migrants  -Looked at various migration variables |

| **Question** | **Response** |
| --- | --- |
| **Study Characteristics** | |
| **Title** | Uninsured immigrant and refugee children presenting to canadian paediatric emergency departments: Disparities in help-seeking and service delivery |
| **Journal** | Paediatrics & Child Health |
| **Authors and Year of Publication** | Rousseau, C., Laurin-Lamothe, A., Rummens, J.A., Meloni, F., Steinmetz, N., & Alvarez, F. (2013) |
| **Study Objectives** | To explore possible differences in help-seeking and service delivery across migratory statuses, institutions and provinces. |
| **Study Design** | Retrospective cohort |
| **City and Province** | Montreal (Quebec) and Toronto (Ontario) |
| **Data Collection Time Frame** | 2008 to 2009 |
| **Follow-Up Time Period** | NR |
| **Data Collection Methods** | -Chart review of patient records from 2008-2009 from 3 hospitals  -Charts were randomly sampled from a curated list of uninsured files. Files were randomly selected from hospitals that had many files. In hospitals with few files of children without OHIP or RAMQ, all files were included in list |
| **Population Characteristics** | |
| **Total Sample Size** | n= 2035  Refugee claimants (federally insured) = 1186 (55.9%)  Uninsured = 836 (39.4%) [people without papers=809, permanent resident during waiting period=17, refused refugee status=4, people between two statuses=3, temporary foreign worker=2, temporary suspension of removal=1]  Visitors=89 |
| **Comparator / Control Group** | Refugee claimants (federally insured) = 1186 (55.9%) |
| **Sample Selection Methods** | Uninsured children were selected using a two step process:   - All children without OHIP or RAMQ in the two year study period were identified. In hospitals with many files, a subset was randomly selected. In hospitals with few, all files were reviewed. Medical charts from the 3 hospitals for 2008-2009 were obtained (one of the hospitals had electronic files). - Children without RAMQ/OHIP were randomly selected from the list of patients without insurance. |
| **Uninsured Definition** | Uninsured is defined as "no access to coverage whatsoever" meaning no access to provincial health insurance and no access to coverage provided by the federal government. This includes:   - Undocumented & grey zone children: Do not have federal or provincial health care coverage. - New permanent resident children: 3 month waiting period for provincial health care coverage. |
| **Sex Distribution (% Male)** | NR |
| **Sex Distribution (% Female)** | NR |
| **Age Distribution** | NR |
| **Ethnicity / Source Country** | NR |
| **Other Baseline Demographics or Characteristics** | NR |
| **Participant Eligibility Criteria** | -Children 0-18 years of age presenting in the Emergency Department.  -Must be:   - Covered by the IFHP - Undocumented or having no insurance coverage from the federal or provincial government - New permanent resident in 3 month waiting period for OHIP.   -Excluded files for non-migrant, out-of-province or forgotten/lost card clients |
| **Results: Cost** | |
| **Were cost-estimates provided for the patient population / individual studied?** | No |
| **Results** | NR |
| **Results: Health Outcomes** | |
| **Was being medically insured studied as a risk factor for negative health outcomes?** | Yes |
| **If poor health outcomes were reported, what demographic characteristics were studied as risk factors?** | NR |
| **What health outcomes were reported in the medically uninsured population?** | -Triage level of emergency care  -Medical and social problems reported  -Treatment and follow-up |
| **Results** | Compared to federally insured children, uninsured children presented more often for:  -Musculoskeletal injuries or lacerations (12.1% in refugee and 20.7% in uninsured; P<0.001), depression (0.4% in refugee and 3% in uninsured; P<0.001), post-traumatic stress disorder (0% in refugee and 0.4% in uninsured; P<0.001), suicidal thoughts (0.8% in refugee and 2.3% in uninsured; P=0.008) or substance abuse (0.2% in refugee and 0.9% in uninsured; P=0.048)  Compared to uninsured children, refugee claimant children were more frequently:  -Diagnosed with respiratory virus infection (30.2% in refugee and 23.4% in uninsured; P=0.001), abdominal pain (4% in refugee and 2.3% in uninsured; P=0.035), sickle cell anaemia (3.5% in refugee and 1.4% in uninsured; P=0.005) and appendicitis (1.3% in refugee and 0.2% in uninsured; P=0.009). Not-significant differences were gastroenteric virus (9.6% in refugee and 7.7% in uninsured), bacterial infection (6.1% in refugee and 6.3% in uninsured), eczema/rash (3.4% in refugee and 2.4% in uninsured), asthma (1.8% in refugee and 1.6% in uninsured), behavioural problems (opposition, relational problems 1.6% in refugees and 2.5% in uninsured), pervasive developmental disorder (1.5% in refugee and 0.8% in uninsured), and negligence (0.1% in refugee and 0.1% uninsured) |
| **Utilization Outcomes** | |
| **What healthcare service was accessed by the medically uninsured population / individual?** | -Emergency room use |
| **Was the number / amount of times that the service was accessed reported?** | No |
| **Was the duration of healthcare utilization reported?** | No |
| **Results** | In total, uninsured children were more likely to present at hospitals with a higher "level of emergency” (denoted 1-5 by the authors with 1=highest, 5=lowest) compared to refugee children insured under the IFHP. Specifically, 1.3% of uninsured children compared to 0.3% of refugee children had the highest level of emergency (1) (χ2= 15,290.01; P<0.001) and 11.5% of uninsured children compared to 8.5% of refugee children had the second highest level of emergency (2) (χ2= 89,055.93;P<0.001). At lower levels of emergency (3-5), differences were not significant between groups. Going from highest level of emergency (1) to lowest level (5), 1= 1.3% uninsured (compared to 0.3%), 2=11.5(8.5), 3=33.4%(31.9%), 4=43.5%(48.1%), 5=10.2%(11.1%). In hospitals 2 (Montreal) (t=4.81; P<0.001) and 3 (Toronto) (t=6.83; P<0.001) the mean emergency rating at triage for uninsured immigrant and refugee claimant children was significantly higher (less urgent) than the mean emergency rating of the overall hospital populations. In contrast, in hospital 1 (Montreal), the refugee claimant and uninsured children status mean emergency rating was comparable with the overall hospital population mean emergency rating (t=−1.62; P=0.105). Hospitalization of refugee claimants was more frequent in hospital 1 (25.1%) in Montreal compared to hospital 2 (2.5%) in Montreal and hospital 3 (9.2%) in Toronto [ p<0.001]. There were differences between hospitals in medication prescription and being able to see a doctor. Hospitalization rate was in: hospital 1, 25.1% for refugees and 12.9% for uninsured; hospital 2, 2.5% for refugees and 3.9% for uninsured; hospital 3, 9.2% for refugees and 9.6% for uninsured. Medication rate was in: hospital 1, 31.7% for refugees and 38.8% for uninsured; hospital 2, 82.3% for refugees and 82.9% for uninsured; hospital 3, 63.2% for refugees and 43.6% for uninsured. Leave without follow-up was in: hospital 1, 22.6% for refugees and 22.3% for uninsured; hospital 2, 1.9% for refugees and 1.4 % for uninsured; hospital 3, 15.3% for refugees and 11.9% for uninsured. Leave with follow-up was in: hospital 1, 20.2% for refugees and 25.2% for uninsured; hospital 2, 0.9 % for refugees and 3.2 % for uninsured; hospital 3, 8.0% for refugees and 32.6% for uninsured. Leave without seeing doctor was in: hospital 1, 0.4 % for refugees and 0.7% for uninsured; hospital 2, 12.4% for refugees and 8.5 % for uninsured; hospital 3, 4.3% for refugees and 2.3% for uninsured. |
| **Conclusion** | -Uninsured children were overrepresented in the very urgent triage category and presented more often for injuries, trauma and mental health problems than did refugee claimant children (covered by IFHP).  -The authors interpreted this overrepresentation as uninsured populations may present to the emergency department only when there are no other options available. |
| **Limitations** | -Due to the retrospective chart review design, socio demographic variables were unavailable or missing and therefore could not be accounted for.  -No control for confounding effects (multivariate regression).  -Potential differences across hospitals were not studied.  -Retrospective chart reviews can be problematic due to inaccuracy and inconsistency. For example, responses may have been influenced by knowledge of immigrant status or different chart records across hospitals (one was electronic while others were not). |
| **Strengths** | -Large total sample size.  -Provided important information on the differences seen in children insured by the IFHP and uninsured children. |

| **Question** | **Response** |
| --- | --- |
| **Study Characteristics** | |
| **Title** | Perinatal outcomes of uninsured immigrant, refugee and migrant mothers and newborns living in Toronto, Canada |
| **Journal** | International Journal of Environmental Research and Public Health |
| **Authors and Year of Publication** | Wilson-Mitchell, K., & Rummens, J. A. (2013) |
| **Study Objectives** | To explore the relationship between insurance status and various perinatal outcomes. |
| **Study Design** | Retrospective cohort study |
| **City and Province** | Toronto, ON |
| **Data Collection Time Frame** | 2007 to 2010 |
| **Follow-Up Time Period** | NR |
| **Data Collection Methods** | -Chart review of hospital records of mothers and newborns in two Toronto community hospitals.  -Insured patients were randomly selected.  -Uninsured patients were not randomly selected (selection method not described) |
| **Population Characteristics** | |
| **Total Sample Size** | n= 453  Uninsured= 175  Insured= 278 |
| **Comparator / Control Group** | Insured= 278  The study’s insured population sample consisted of all pregnant women, regardless of place of birth or citizenship status, who had a valid provincial OHIP card. These included both Canadian-born and landed immigrant women, as well as temporary workers and visitors with some type of temporary OHIP coverage such as farm workers, individuals on work permits, graduate students, clergy and missionaries. |
| **Sample Selection Methods** | -The insured group was randomly generated from the hospital's list of OHIP patients during the four year study period  -The uninsured group was not randomly selected. 325 charts were obtained and 150 were excluded. The sample was generated from hospital record lists using self-pay payment codes. Three groups were excluded: refugee claimants covered under IFHP, individuals with coverage from another province, people with private insurance or homeless women. |
| **Uninsured Definition** | "Pregnant women seeking care who did not have OHIP, IFHP or private health insurance." At the time of the study these included: (i) new immigrant permanent residents within the three month waiting period for provincial health care coverage, as well as those currently applying for landed immigrant status through their spouse; (ii) successful refugee claimants not covered by the Interim Federal Health Programme benefits, asylum-seekers awaiting decision regarding their refugee claim, and those whose claims have been denied; and (iii) undocumented or partially documented migrants who have outstayed a visitor’s visa or work permit, or who have entered the country through non-regular means. |
| **Sex Distribution (% Male)** | 0 (0%) |
| **Sex Distribution (% Female)** | 453 (100%) |
| **Age Distribution** | Insured mean= 29.3  Insured mode= 30  Uninsured mean= 28.6  Uninsured mode= 27 |
| **Ethnicity / Source Country** | Uninsured:  African (7.1%), South Asian (10.3%), Middle Eastern (9.6%), Eastern American (2.8%), Western American (3.2%), South American (0.6%), Central American (2.6%), Caribbean (40.4%), East Asian and Pacific (9.6%), American (1.3%), Canadian (0.6%), Other (11.5%)  Insured:  African (5.6%), South Asian (25.7%), Middle Eastern (2.8%), Eastern American (2.8%), Western American (3.2%), South American (0.6%), Central American (0%), Caribbean (12.8%), East Asian and Pacific (8.4%), American (0%), Canadian (4.5%), Other (30.2%) |
| **Other Baseline Demographics or Characteristics** | -Ethnicity  -Age  -Primigravidas  -Community health centre use  -Male infant births  -Mean gestational age  -Gestational diabetes  -Chronic hypertension  -Smoking |
| **Participant Eligibility Criteria** | -Having given birth at one of the 2 Toronto Ontario hospitals within the date ranges  -Exclusion criteria: those using IFHP, individuals with coverage from another province, homeless women, women with pre-pregnancy or pre-existing high risk medical conditions to ensure the two groups were comparable |
| **Results: Cost** | |
| **Were cost-estimates provided for the patient population / individual studied?** | No |
| **Results** | NR |
| **Results: Health Outcomes** | |
| **Was being medically insured studied as a risk factor for negative health outcomes?** | Yes |
| **If poor health outcomes were reported, what demographic characteristics were studied as risk factors?** | -Uninsured status  -Smoking status |
| **What health outcomes were reported in the medically uninsured population?** | -Caesarean section rate, maternal complications, neonatal complications, low birth weight, small for gestational age, preterm birth, Neonatal Intensive Care Unit (NICU) admission, and exclusive breastfeeding upon discharge |
| **Results** | -No significant differences were found between the insured and uninsured groups with respect to low birth rates (5.71%(2.28-9.15) in uninsured vs. 7.97%(4.78-11.17) in insured; not significant), preterm birth rates (7.43%(3.54-11.31) vs. 8.27% (5.04-11.51); not significant), breastfeeding rates (91.02% (86.68-95.35) vs. 86.45(82.39-90.51); not significant)  -More caesarean sections occurred in the insured group (35.6%) than in the uninsured group (26.3% with Pearson’s χ2 = 4.292, α = 0.038). Reasons for this differed between groups, most common reason among insured women was labor dystocia, most common reason among uninsured was abnormal fetal heart rate (35% versus 21.7%, with χ2 = 5.405, α = 0.020)  -Exclusive breastfeeding was continued at discharge by 91% of the uninsured and 86.4% of the insured upon discharge  -The newborns of uninsured mothers had a significantly higher incidence (9.7% versus 4.3% with χ2= 5.174, α = 0.023) of major resuscitation involving positive pressure ventilation and/or heart compressions than did those of insured mothers  -Both the uninsured and insured groups had higher than expected rates of postpartum hemorrhage (PPH), 3.8% and 4.0% respectively |
| **Utilization Outcomes** | |
| **What healthcare service was accessed by the medically uninsured population / individual?** | -Number of prenatal visits, provider type, length of hospital stay |
| **Was the number / amount of times that the service was accessed reported?** | Yes |
| **Was the duration of healthcare utilization reported?** | Yes |
| **Results** | -6.5% of uninsured women received no prenatal care at all, 100% of insured women had care  -The number of prenatal visits reported for the uninsured group (mean = 6.04 (5.40-6.68) was significantly lower than the insured group (8.70(8.35-9.05; p=0.001)  -The uninsured pregnant women sought the services of midwives significantly more than did the insured, 36.3% versus 4.0%. 55.4% of uninsured saw an OB, 94.1% of insured saw an OB. An equal percentage saw a GP (1.8%). 6.5% of the uninsured reported no health care provider while 0% of the insured did.  -53.7% of the uninsured women had received 'clearly inadequate prenatal care', compared to 19.6% insured women. Insured women had comparatively higher levels of intermediate care (six to eight visits); and uninsured women had significantly higher rates of clearly inadequate care (zero to five visits). Insured with OHIP: 3.8% had inadequate care (0-2 visits), 15.8% inadequate (3-5), 44.2% intermediate (6-8), 28.8% adequate (9-12), 7.3% adequate plus (13 or more visits). Uninsured: 32.2% had inadequate care (0-2 visits), 21.5% inadequate (3-5), 27.5% intermediate (6-8), 12.1% adequate (9-12), 6.7% adequate plus (13 or more visits).  -Newborns of uninsured mothers had a significantly higher incidence (9.7% versus 4.3% with χ2= 5.174, α = 0.023) of neonatal resuscitation. The difference in NICU admission was not significant (15.16% insured (10.94-19.39), 14.37 (9.16-19.58)% uninsured).  -C-Sections occurred more in the insured group (35.6%) compared to uninsured (26.3%) (χ2 = 4.292, α = 0.038) but uninsured women had a significantly higher rate of caesarean sections due to abnormal fetal heart rate (35% versus 21.7%, with χ2= 5.405, α = 0.020)  -Length (in days) of hospital stay was significantly less for the uninsured mothers (1.65 (1.49-1.82) versus 2.3 (2.19-2.46)) while length of hospital stay for baby was 2.05 (1.54-2.55) in uninsured and 2.14 (2.00-2.29) in insured |
| **Conclusion** | -6.5% of uninsured women received no prenatal care at all.  -Insurance status was related to the type of health care provider and reason for caesarean section.  -Uninsured women had shorter maternal length of hospital stay.  -Uninsured mothers experienced a higher percentage of cesarean sections due to abnormal fetal heart rates and required more neonatal resuscitations.  -No significant difference was found for low birth weight, preterm birth, NICU admissions, postpartum hemorrhage, breast feeding, or intrapartum care provided |
| **Limitations** | -Information regarding place of birth and length of Canadian residency is not included in the charts  -Retrospective chart reviews are problematic due to inaccuracy and inconsistency  -Researchers were not able to match cases on relevant demographic information because this information was inconsistently recorded  -Small sample size decreased the power  -The findings are not generalizable |
| **Strengths** | -Provides evidence for health policy analysts and decision makers |

| **Question** | **Response** |
| --- | --- |
| **Study Characteristics** | |
| **Title** | Cervical cancer screening among vulnerable women |
| **Journal** | Canadian Family Physician |
| **Authors and Year of Publication** | Wiedmeyer, M., Lofters, A., & Rashid, M. (2012) |
| **Study Objectives** | To see if refugee women at a community health centre (CHC) in Toronto, Canada are appropriately screened for cervical cancer and if there are any demographic characteristics that affect whether they are screened. |
| **Study Design** | Retrospective cohort |
| **City and Province** | Toronto, ON |
| **Data Collection Time Frame** | January 1, 2004 to September 1, 2008 |
| **Follow-Up Time Period** | N/A |
| **Data Collection Methods** | -Chart review of all patient records from the community health centre from 2004-2008 (sampling not necessary)  -Database search of all registered clients of Access Alliance Multicultural Health and Community Services meeting criteria within the specified timeline |
| **Population Characteristics** | |
| **Total Sample Size** | n= 357  Insured= 274  Uninsured= 83 |
| **Comparator / Control Group** | Insured n= 274 |
| **Sample Selection Methods** | Database search of all registered clients of AAMHCS meeting criteria within the specified timeline. |
| **Uninsured Definition** | NR |
| **Sex Distribution (% Male)** | N/A |
| **Sex Distribution (% Female)** | n= 357 (100% Female) |
| **Age Distribution** | Age 18 to 69 at time of registration but not further specified |
| **Ethnicity / Source Country** | Country of origin was classified as European or Non-European but not further specified. |
| **Other Baseline Demographics or Characteristics** | -Immigration status  -Insurance status  -Year of arrival  -Country of origin  -Preferred language  -Documentation of reason for no Pap test, if none  -Pap test result  -Follow-up  -Pregnancy |
| **Participant Eligibility Criteria** | -Women aged 18 to 69 at registration  -Enrolled between January 1, 2004 to September 1, 2008  -Who had received at least 1 visit with a physician at AAMHCS, as opposed to only seeing an allied health professional  -Who had received at least 3 visits to the clinic during the study period |
| **Results: Cost** | |
| **Were cost-estimates provided for the patient population / individual studied?** | No |
| **Results** | N/A |
| **Results: Health Outcomes** | |
| **Was being medically insured studied as a risk factor for negative health outcomes?** | No |
| **If poor health outcomes were reported, what demographic characteristics were studied as risk factors?** | N/A |
| **What health outcomes were reported in the medically uninsured population?** | N/A |
| **Results** | N/A |
| **Utilization Outcomes** | |
| **What healthcare service was accessed by the medically uninsured population / individual?** | -Pap smear |
| **Was the number / amount of times that the service was accessed reported?** | NR, however 284/357 charts reported having a Pap smear documented in the chart, 75% of insured women had a Pap smear and 95% of uninsured women had a Pap smear. |
| **Was the duration of healthcare utilization reported?** | No |
| **Results** | -Univariate analysis: women without insurance were significantly more likely to have Pap tests than those who were insured (odds ratio 6.65, P < .0001)  -Controlling for the potential confounders of language, region of origin, year of arrival, pregnancy, and age, the association dropped and became non-significant (OR=2.710, 95% CI=0.797-9.259) during logistic regression. Speaking English and region of origin were not significantly correlated with having a Pap test  -In the cox proportional hazard model, insurance status was significantly associated with time to first pap test (adjusted hazard ratio=1.715, 9% CI=1.156-2.545). Although uninsured patients were more likely to get a Pap test at any point in time in this model, after adjustment for all main regions of origin, rather than simply using the stratification of European versus non-European, this result became non-significant (adjusted hazard ratio 1.312, 95% CI 0.922 to 2.058)  -Speaking English significantly predicted the likelihood of getting a Pap test earlier after registration, relative to non-English speakers (adjusted hazard ratio 0.625 95% CI 0.462–0.854) |
| **Conclusion** | -Most refugee and uninsured clients at AAMHCS were appropriately screened for cervical cancer.  -92% of women were approached for a Pap test, and 80% of women had a Pap test done during the study period.  -There was no demographic characteristic that significantly predicted a missed Pap test. The only significant variable affecting Pap testing was the inability to speak English.  -Although being uninsured was associated with a significantly higher likelihood of receiving a Pap test in univariate analysis, multivariate analyses suggest that this relationship was confounded by other variables, such as pregnancy and speaking English, among the uninsured patients. Rates of pregnancy are high among the uninsured population at AAMHCS, and many of the uninsured are from the Caribbean region and are therefore English speakers. |
| **Limitations** | -Small sample size and lack of statistical power.  -All data were from one clinic so the results are not generalizable to other locations.  -Did not analyse provider effects such as male sex of the physician, comfort with sexual history taking, or practice differences between nurse practitioners and physicians. |
| **Strengths** | -Strengths of this study are the multivariate analysis of significant demographic variables and access to a concentrated population of refugees. |

| **Question** | **Response** |
| --- | --- |
| **Study Characteristics** | |
| **Title** | Retrospective review of prenatal care and perinatal outcomes in a group of uninsured pregnant women |
| **Journal** | Journal of Obstetrics and Gynaecology Canada |
| **Authors and Year of Publication** | Jarvis, C., Munoz, M., Graves, L., Stephenson, R., D’Souza, V., & Jimenez, V. (2011) |
| **Study Objectives** | To assess prenatal and perinatal health outcomes, specifically adequacy of care, among uninsured pregnant women in Montreal, Canada. |
| **Study Design** | Retrospective cohort study |
| **City and Province** | Montreal, QC |
| **Data Collection Time Frame** | 2004 to 2007 |
| **Follow-Up Time Period** | NR |
| **Data Collection Methods** | -Chart audit conducted at the Herzl Family Practice Centre and the Centre Local de Services Communautaires Cote des Neiges in Montreal, Canada using medical databases  -Chart audit form developed to collect demographic information, information on legal status in Canada (if available), insurance status, number of prenatal visits, routine prenatal investigations, and basic perinatal outcomes  -Random sampling to obtain insured cohort |
| **Population Characteristics** | |
| **Total Sample Size** | n= 143  Uninsured=71  [Visitor or tourist=5; foreign student=2; awaiting sponsorship=8; undocumented immigrants=41; asylum seekers who have not yet filed a claim=5; refused refugee claimants=7]  Insured= 72 |
| **Comparator / Control Group** | Provincially insured women (n=72) |
| **Sample Selection Methods** | -Convenience sampling (uninsured cohort) and random sampling (insured cohort).  -A retrospective chart audit was conducted at the Herzl Family Practice Centre and Centre Local de Services Communautaires Cote des Neiges in Montreal. Women presenting for prenatal care (n=1523) at the two centres between 2004 and 2007 were selected for the study using the databases and monthly obstetric rosters.  -From these records, women who did not have insurance at initial presentation (n=71) were chosen to form the study cohort.  -Control group (n=72) was randomly selected from provincially insured women presenting for prenatal care between 2004 and 2007. |
| **Uninsured Definition** | -Uninsured is defined as individuals living in Canada who are not eligible for government insurance. This can include undocumented (illegal) immigrants, as well as individuals with precarious status such as "tourists, visitors, foreign students, undocumented asylum seekers, and persons who are between different status types." |
| **Sex Distribution (% Male)** | 0 (0%) |
| **Sex Distribution (% Female)** | 143 (100%) |
| **Age Distribution** | Insured mean= 29.2  Uninsured mean= 28.6 |
| **Ethnicity / Source Country** | NR |
| **Other Baseline Demographics or Characteristics** | -Maternal age years  -Gravidity  -Parity |
| **Participant Eligibility Criteria** | -Pregnant women who visited Herzl Family Practice Centre and Centre Local de Services Communautaires Cote des Neiges for prenatal care between 2004 and 2007 in Montreal  -For uninsured women no insurance was recorded at initial presentation at the centres |
| **Results: Cost** | |
| **Were cost-estimates provided for the patient population / individual studied?** | No |
| **Results** | N/A |
| **Results: Health Outcomes** | |
| **Was being medically insured studied as a risk factor for negative health outcomes?** | Yes |
| **If poor health outcomes were reported, what demographic characteristics were studied as risk factors?** | -Maternal age years  -Gravidity  -Parity |
| **What health outcomes were reported in the medically uninsured population?** | -Perinatal outcomes assessed gestational age and birth weight of insured and uninsured participant's baby |
| **Results** | -Uninsured women had lower gestational age at birth (39.0 weeks compared to 39.2 weeks for insured women) and baby’s had lower weight at birth (3,379g compared to 3,387g for insured women) |
| **Utilization Outcomes** | |
| **What healthcare service was accessed by the medically uninsured population / individual?** | -Routine prenatal services (including blood tests, obstetric ultrasound, cervical swab for STIs, Pap tests and early genetic screening)  -Postpartum services  -Visits with healthcare provider  -Route of delivery (vaginal birth, caesarean section) |
| **Was the number / amount of times that the service was accessed reported?** | Yes |
| **Was the duration of healthcare utilization reported?** | Yes |
| **Results** | -Uninsured women had fewer initial screening blood tests conducted (93.7% vs. 100%; p=0.045), ultrasound screenings (82.5% vs. 98.4%; p=0.003), cervical swabs (69.8% vs. 85.2%; p=0.04), Pap tests (38.1% vs. 75.4%; p<0.001), genetic screenings (12.7% vs. 44.3%; p<0.001), lower mean total number of prenatal visits (6.6 (3.4 SD) vs. 10.7(3.0 SD); p=0.05), and less physical examinations (6.6% vs. 10.7%; not statistically significant)  -Vaginal birth was higher in uninsured women (71.4% vs. 69.5%; not significant) and caesarean sections were lower (28.6% vs. 30.5%; not significant). In terms of delivery interventions, epidural use was lower among uninsured (71.4% vs. 73.3%; not significant) and induction was higher (25.4% vs. 20%; not significant)  -Gestational age at birth was 39(1.8) for uninsured women and 39.2(1.4) for insured women (not statistically different from one another), and baby's weight at birth was 3379(550) for uninsured and 3387 for insured (not significant).  -Gestational age at first visit for insured women was 25.6weeks (11.1) and 12 (5.7) for insured women (p<0.001)  -Using the Prenatal Care Utilization Index, in the uninsured group, 61.9%, 12.7%, 20.6%, and 4.8% had inadequate, intermediate, adequate, and adequate plus prenatal care utilization. In the insured group, 11.7%, 13.3%, 55%, and 20% had inadequate, intermediate, adequate, and adequate plus prenatal care utilization. The difference between the group was significant at X2=36.3 and p=0.001.  -In terms of adequacy of received services, 6.3%, 33.3%, 44.4%, and 15.9% had inadequate, intermediate, adequate, or adequate plus respectively. For insured it was 1.7%, 15%, 58.3%, and 25% respectively. The difference was statistically significant with X2=8.3 and p=0.04.  -Uninsured pregnant women presented for initial care 13.6 weeks later than insured women (at 25.6 weeks vs. 12.0 weeks, P < 0.001) |
| **Conclusion** | -It was found that uninsured women had fewer routine prenatal screening tests, ultrasound examinations, swabs for STI, Pap tests, and genetic screening than women with insurance. This difference in rates of screening may be attributed to financial challenges such as covering for the cost of testing among uninsured women  -The uninsured women in the study presented late for prenatal care and were less likely to receive both routine prenatal screening tests and adequate prenatal care compared to insured women. |
| **Limitations** | -Difficult population to study as uninsured are often undocumented  -This study is not representative of uninsured women with no prenatal care. Results may under-represent the magnitude of the issue as women with no care tend to have worse outcomes (therefore results may not be generalizable)  -One of the family health centers provided financial assistance to women, undermining the initial objective of assessing women that are completely uninsured  -Due to the nature of the study, it was difficult to collect socio demographic information. Confounders may have been missed. |
| **Strengths** | -Less resource intensive and the study is relatively less expensive to conduct  -Effect size calculation was performed (Accumulated data from a large number of patients)  -Uninsured group is broad and includes undocumented, travelers, students and asylum seekers |
